# Grapevine Virome of the Don Ampelographic Collection in Russia Has Concealed Five Novel Viruses

**DOI:** 10.3390/v15122429

**Published:** 2023-12-14

**Authors:** Daria Belkina, Daria Karpova, Elena Porotikova, Ilya Lifanov, Svetlana Vinogradova

**Affiliations:** 1 Skryabin Institute of Bioengineering, Research Center of Biotechnology of the Russian Academy of Sciences, Leninsky Prospect, 33, Build. 2, 119071 Moscow, Russia; daria.microbiology@yandex.ru (D.B.);; 2 North Caucasian Federal Scientific Center of Horticulture, Viticulture, Wine-Making, 40 Years of Victory Street, Build. 39, 350901 Krasnodar, Russia

**Keywords:** *Vitis vinifera*, grapevine virome, RNA-seq, umbra-like virus, alphapartitivirus, secovirus, grapevine viruses, grapevine germplasm, high-throughput sequencing

## Abstract

In this study, an analysis of the virome of 51 grapevines from the Don ampelographic collection named after Ya. I. Potapenko (Russia) was performed using high-throughput sequencing of total RNA. A total of 20 previously described grapevine viruses and 4 viroids were identified. The most detected were grapevine rupestris stem pitting-associated virus (98%), hop stunt viroid (98%), grapevine Pinot gris virus (96%), grapevine yellow speckle viroid 1 (94%), and grapevine fleck virus (GFkV, 80%). Among the economically significant viruses, the most present were grapevine leafroll-associated virus 3 (37%), grapevine virus A (24%), and grapevine leafroll-associated virus 1 (16%). For the first time in Russia, a grapevine-associated tymo-like virus (78%) was detected. After a bioinformatics analysis, 123 complete or nearly complete viral genomes and 64 complete viroid genomes were assembled. An analysis of the phylogenetic relationships with reported global isolates was performed. We discovered and characterized the genomes of five novel grapevine viruses: bipartite dsRNA grapevine alphapartitivirus (genus *Alphapartitivirus*, family *Partitiviridae*), bipartite (+) ssRNA grapevine secovirus (genus *Fabavirus*, family *Secoviridae*) and three (+) ssRNA grapevine umbra-like viruses 2, -3, -4 (which phylogenetically occupy an intermediate position between representatives of the genus *Umbravirus* and umbravirus-like associated RNAs).

## 1. Introduction

The grapevine is one of the most important and widespread cultivated plants; the history of its cultivation goes back thousands of years. According to the International Organization of Vine and Wine, vineyards occupy more than 7 million hectares of the Earth’s surface, about 33 million tons of wine and 30 million tons of table grapes are produced annually [1]. In 2022, vineyards in Russia occupied 101 thousand hectares. In terms of the area of vineyards, the country ranks 19th in the world [2]. The viticulture and winemaking industries are actively developing in the south of Russia.

However, globalization and climate change have negatively affected the health of grapevines, with pathogens spreading along with infected planting material and new diseases appearing in changing environmental conditions. Grapevine viruses pose a serious threat to grapevine plantations around the world. To date, more than 100 viral pathogens of grapevine are known [3,4,5,6,7,8,9,10,11,12,13]. About half of them can cause diseases that are usually associated with rugose wood complex, leafroll, leaf degeneration, and fleck disease [14]. The effects of many viruses on the grapevine are still poorly understood. Some viruses are known to be neutral towards their host, but when environmental conditions change, latent infection can develop into a serious disease [15].

In recent years, high throughput screening (HTS) technologies have opened up new perspectives in phytosanitary research. Due to the fact that grapevine is the only agricultural plant affected by such a large number of viruses, HTS methods are the most suitable for detecting viral pathogens and are successfully used to study their genomes, genetic diversity, and evolution, and also make it possible to detect and characterize previously unknown viruses [16,17].

Currently, there are no effective methods to cure plants of viral diseases; therefore, their timely monitoring and the use of healthy planting material are very important [18]. Monitoring is of particular importance when it comes to grapevine germplasm. Ampelographic collections are necessary for the preservation of genetic resources of grapevine, the selection of new cultivars, and the classification of existing ones. However, long-term maintenance of cultivars inevitably leads to the accumulation of viral pathogens, which jeopardizes the selection efforts and reduces the value of the cultivars maintained in the collection.

The problem of virus accumulation in the grapevine germplasm collections is being studied worldwide. For example, screening of autochthonous cultivars from the Balearic Islands (Spain) has shown that more than 52% of the grapevines are affected by a mixed viral infection, with the economically significant GLRaV-3 and GFLV being some of the most common viruses [19]. A metaviromic study of betaflexiviruses in the South African ampelographic collection has also revealed a predominance of multiple virus infections, including economically significant viruses [20].

In Russia, ampelographic collections are concentrated in the southern regions. Previously, studies of the virome of grapevine from the collections of the Krasnodar Krai [21] and the Republic of Dagestan were carried out [22]. As a result, valuable information was obtained on the distribution of viral pathogens in these collections. In the Dagestan collections, about 70% of the analyzed plants were infected by the grapevine fanleaf virus, one of the most harmful grapevine viruses [23]. For the first time in Russia, it was detected grapevine virus B (GVB), grapevine virus F (GVF), grapevine asteroid mosaic-associated virus (GAMaV), grapevine Red Globe virus (GRGV), grapevine satellite virus (GV-Sat), Vitis cryptic virus (VCV), grapevine leafroll-associated virus 7 (GLRaV-7), and grapevine Kizil Sapak virus (GKSV). In addition, in these ampelographic collections were discovered and described two novel grapevine viruses: (+) ssRNA grapevine umbra-like virus (GULV) from the family *Tombusviridae* and dsDNA grapevine pararetrovirus (GPRV) from the family *Caulimoviridae* [21,22].

The virome of one more ampelographic collection, the Don ampelographic collection, named after Ya. I. Potapenko, was analyzed in this study. Its distinctive feature is that it is located in the zone of covered industrial viticulture in the Rostov Oblast of Russia and comprises about 828 grape cultivars and hybrids from more than 40 countries [24]. Among them, there are both widely cultivated in the world cultivars and autochthonous Caucasian cultivars [25], as well as less common cultivars of local selection that are promising for winemaking in covered viticulture conditions [26]. Most of the accessions in the collection come from Russia (40%), Moldova (7%), Uzbekistan (5%), France (5%), Georgia (5%), Ukraine (4%), Hungary (4%), USA (4%), and Armenia (4%) [24].

In this article, the results of a study of the grapevine virome of the Don ampelographic collection that was performed using high-throughput sequencing technologies are presented.

## 2. Materials and Methods

### 2.1. Plant Material and Preparation of mRNA Libraries

In September 2019, phytosanitary monitoring of the Don ampelographic collection, named after Ya. I. Potapenko in the Rostov Oblast was carried out, collecting 51 samples (Supplementary Table S1) with symptoms of a viral infection to extract total RNA and prepare libraries for sequencing. A 1 g sample of grapevine leaf petioles and veins was used as the material for the RNA extraction. RNA was extracted according to the method of Morante-Carriel et al. [27], using CTAB buffer as a lysis solution and 10 M LiCl for the RNA purification and reprecipitation step. The quality of the RNA extraction and concentration were verified using an Eppendorf BioSpectrometer and electrophoresis in 1% agarose gel.

Extracted RNA samples were used for library preparation after their pretreatment with DNase I (Thermo Fisher Scientific, Waltham, MA, USA) according to the manufacturer’s protocol. Each library represented a single grapevine plant. Processed samples were depleted of rRNAs by removing the rRNA-probe complex with magnetic bead technology using the RiboMinus Plant Kit (Thermo Fisher Scientific, Waltham, MA, USA). The concentration of the obtained samples was measured on a Qubit 4.0 fluorimeter (Invitrogen, Waltham, MA, USA) using the Qubit RNA BR Assay Kit (Thermo Fisher Scientific, Waltham, MA, USA). Library preparation was performed using the QIAseq Stranded RNA Library Kit (Qiagen, Hilden, Germany), their quality was checked on a Qsep1 capillary electrophoresis system (BiOptic, New Taipei, Taiwan), and the DNA concentration was measured using the HS DNA assay kit (Thermo Fisher Scientific, Waltham, MA, USA). The finished libraries were sequenced on a NovaSeq 6000 System (Illumina, San Diego, CA, USA). FASTQ raw sequencing data were deposited to the Sequence Read Archive (SRA) (accession number: PRJNA1043183).

### 2.2. HTS Data Analysis and Assembly of Viral Genomes

Bioinformatics data processing was carried out using the Geneious Prime v. 2022.2.2 program (Biomatters, Ltd., Auckland, New Zealand). Preprocessing included trimming of paired reads with trimming of adapters and low-quality reads using the BBDuk plugin (minQ = 30, min length = 10 bp). Then, the reads were merged, and duplicates were removed (kmer = 30).

*De novo* assembly was carried out using the SPAdes (default settings) and Geneious assemblers (medium-low sensitivity). Contigs were compared to the NCBI reference viral genome database (accessed on 15 September 2022) using the tblastx algorithm. The results were displayed in a hit table, with a max E-value of 0.05. For further analysis, contigs of plant viruses with E-value ≤ 1 × 10^−40^ were selected. In parallel with *de novo* assembly, preprocessed reads were mapped with medium-low sensitivity (maximum mismatches per read 20%) to the reference genomes of grapevine viruses and viroids (accessed on 24 June 2023).

In the analysis of the viruses GLRaV-1, GLRaV-2, GLRaV-3, GRSPaV, GFLV, GPGV, GVA, GVB, and GVF with high coverage (more than a thousand reads per reference genome), a threshold of 10 reads and 15% of Ref Seq was set. For other viruses, no threshold was applied. Samples confirmed by both bioinformatics analysis of sequencing data and PCR were considered positive.

To assemble complete viral genomes, mapping settings (selection of a reference genome, maximum mismatches per read) were selected so as to achieve a minimum number of ambiguities with maximum coverage. The closest genome for mapping was determined using a blastn analysis of contigs or consensus against the GenBank database. As complete viroid genomes, whole genome contigs assembled by the Geneious assembler with a threshold E-value ≤ 1 × 10^−10^ were taken. Genomes of viruses and viroids were uploaded to the GenBank (Supplementary Table S2) if they covered more than 90% of the reference sequence and had ambiguities < 5% of the consensus length.

Plant virus contigs that matched unexpected grapevine viruses were verified using blastn in the megablast mode against the GenBank database. If blastn did not show a match, it was assumed that the contig belonged to an unknown virus. Reads from all libraries were mapped onto contigs selected in this way. Virus family or order was inferred from nearby genomes using the blastx tool. In cases where we were unable to immediately obtain a contig corresponding to the complete genome of a novel virus, we performed a tblastx analysis of the library contigs against a local database composed of genomes of viruses of the same family or order. Contigs corresponding to plant viruses were verified using the blastn tool. To determine the relative positions of contigs of the novel virus on the genome, the nucleotide sequences were translated into more conservative amino acid sequences and aligned using the Clustal Omega 1.2.2 algorithm with the translated genome sequence of the nearest virus. The gaps between the contigs discovered in this way were closed using Sanger sequencing; the used primers are provided in Supplementary Table S3.

The Step-Out RACE technology was used to determine the sequence of the 5′ and 3′ terminal fragments. Total RNA was used as a template for cDNA synthesis. Using the Mint cDNA synthesis kit (Cat. #SK001, Evrogen, Moscow, Russia), cDNA was amplified using a step-by-step “external priming” method with universal mixtures of adapter primers and then used to synthesize the 5′ and 3′-terminal fragments. Synthesis was carried out using the Encyclo Plus PCR kit (Cat. #PK001, Evrogen, Moscow, Russia) and specially selected gene-specific primers (Supplementary Table S3). Rapid amplification of the 5′ and 3′ terminal fragments of target transcripts was carried out for novel viruses. Reaction conditions were optimized separately for each transcript based on the manufacturer’s protocol.

Analysis of PCR products was carried out by electrophoresis in 1.2% agarose gel. PCR products of the expected size were extracted from the gel using the Cleanup Standard kit (Evrogen, Moscow, Russia) and cloned into the pAL2-T vector using the Quick-TA kit (Cat. #TAK02, Evrogen, Moscow, Russia). Chemical transformation of competent *Escherichia coli* XL1-Blue cells (Cat. #CC001, Evrogen, Moscow, Russia) was carried out with a ligase mixture in accordance with the standard protocol [28]. Then, the *E. coli* cells were cultured on a selective LB medium with ampicillin and the addition of XGal and IPTG. Sequencing of the cloned DNA fragment was carried out using the plasmid primers, M13F and M13R, included in the Quick-TA kit. The resulting nucleotide sequences were assembled using the Geneious program and deposited in the GenBank with accession numbers OR947505-OR947511.

The NCBI ORFfinder tool was used to identify the open reading frames of novel viruses. The InterPro database was used to predict protein domains and functional sites [29].

### 2.3. Validation of Grapevine Viruses and Viroids

All viruses and viroids identified in the samples using bioinformatics methods were validated by RT-PCR and quantitative RT-PCR in each library. Total RNA, random hexamer, and MMLV reverse transcriptase (Evrogen, Moscow, Russia) were used to perform the reverse transcription reaction according to the manufacturer’s protocol. To verify the quality of the reverse transcription reaction, PCR was performed for the 18S rRNA gene, which was used as an endogenous control. RT-PCR was performed using the Encyclo polymerase according to the manufacturer’s protocol (Evrogen, Moscow, Russia). Previously published primers and primers designed using the PrimerBLAST and Beacon Designer Free (Premier Biosoft International, San Francisco, CA, USA) tools were used for virus detection (Supplementary Table S3). PCR products were visualized in a 1.0% agarose gel. All PCR products obtained using newly designed primer pairs were validated with bidirectional Sanger sequencing using the BigDye Terminator v3.1 Cycle Sequencing Kit (Thermo Fisher Scientific, Waltham, MA, USA) on an ABI PRIZM 3730 automated sequencer in accordance with the manufacturer’s instructions. The resulting nucleotide sequences were analyzed using the NCBI BLASTn tool, Finch TV 1.4.0 (Geospiza Inc., Seattle, WA, USA), and MEGA11 [30] programs. All nucleotide sequences were uploaded to the GenBank (Supplementary Table S4).

Validation of HSVd and GYSVd-1 was performed by TaqMan^®^ RT-qPCR using the BioMaster HS-qPCR kit (Biolabmix, Novosibirsk, Russia). The reaction was carried out in three technical replicates. The primers and probes used are listed in Supplementary Table S3. RT-qPCR results were analyzed using the LightCycler 96 SW1.1 software (Roche, Mannheim, Germany). 

### 2.4. Phylogenetic Analysis and Genetic Diversity

Nucleotide or amino acid sequences of viruses available in the GenBank and discovered by us were aligned using the Clustal Omega 1.2.2 method and used for phylogenetic analysis. The sequences were not trimmed. The best model of DNA sequence evolution was searched for using the MEGA11 program [30]. Dendrograms were constructed using the maximum likelihood method in MEGA11. Sequences of the closest species were used as an outgroup. Bootstrap support included 1000 replicates. Information about the type of data, substitution models, outgroups, and number of sequences are shown in Supplementary Table S5.

Pairwise comparisons of viral genome sequences were performed using the Clustal W alignment algorithm in the Sequence Demarcation Tool (SDT) [31].

## 3. Results and Discussion

### 3.1. mRNA-seq Data Analysis

Sequencing 51 total RNA libraries yielded approximately 925 million raw reads. The number of reads for each library ranged from 9 to 32 million, with a mean of 18 million (Supplementary Table S6). As a result of preprocessing, the number of reads was reduced by approximately 2.5 times. All libraries were assembled *de novo* using the SPAdes and Geneious assemblers. After assembly by SPAdes, an average of 4135 contigs per library were obtained, and the N50 length was about 1738 bp. After assembly by Geneious, an average of 87,924 contigs per library were obtained, and the N50 length was about 381 bp (Supplementary Table S6).

### 3.2. Identification of Known Grapevine Viruses and Viroids

#### 3.2.1. Family: *Closteroviridae*

##### Grapevine Leafroll-Associated Virus 1

GLRaV-1 (genus *Ampelovirus*) is an economically important viral pathogen of grapevine and one of the pathogens of leafroll disease (GLD) [32]. In red-berried varieties, additional GLD symptoms include reddening of the interveinal areas of the leaves [33]. Symptoms of GLRaV-1 are enhanced by co-infection with GLRaV-3 [34]. In this study, GLRaV-1 was identified in eight samples (Supplementary Table S7). Mapping of reads to the nearest genomes (Supplementary Table S8) made it possible to obtain two nearly complete genomes of GLRaV-1.

Previously, the genetic diversity of GLRaV-1 was analyzed primarily based on the HSP70 or CP genes [35,36,37]. The number of whole-genome GLRaV-1 sequences in the GenBank is sufficient to construct a phylogenetic tree following Morán et al. [38]. The topology of the tree constructed is consistent with the results of Morán et al. and allows us to divide the isolates into five phylogroups (Supplementary Figure S1). For all nodes, bootstrap support of more than 83 was noted. The isolates obtained in this study were clustered in different groups, which confirms the thesis that there is no association between genetic variants of GLRaV-1 and their geographic origin [36].

##### Grapevine Leafroll-Associated Virus 2

GLRaV-2 (genus *Closterovirus*) is distributed worldwide [39,40,41] and is associated with two types of symptoms: leafroll disease and graft incompatibility [40]. In this study, GLRaV-2 was identified in two samples (Supplementary Table S7). As a result of the mapping of reads to the closest genomes of GLRaV-2 (Supplementary Table S8), one nearly complete genome was obtained.

GLRaV-2 isolates are distinguished by high genetic diversity: based on the analysis of HSP70 and CP genes, they are divided into six phylogenetic groups [41,42]. Viruses from different phylogroups differ in their pathogenic properties [39,40]. The genome with high bootstrap support obtained in this study was found to belong to the BD group (Supplementary Figure S2). There is evidence that isolates from the BD group show low severity of disease symptoms or their complete absence [43,44]. A representative of this phylogroup was reported in the Russian Federation for the first time.

##### Grapevine Leafroll-Associated Virus 3

GLRaV-3 (genus *Ampelovirus*) is one of the most widespread and harmful grapevine viruses [45,46]. It is known as the main pathogen of grapevine leafroll disease (GLD) [47]. GLRaV-3 was identified in 19 samples (Supplementary Table S7). Due to the heterogeneity of the reads, mapping with the option of 5% mismatches per read was performed in order to obtain complete GLRaV-3 sequences (Supplementary Table S8). As a result, 16 complete and nearly complete genomes were obtained.

GLRaV-3 is highly genetically diverse, as indicated by molecular studies of the CP gene [36,48] as well as whole-genome phylogenetic studies [49,50,51]. Currently, genetic variants of GLRaV-3 are divided into nine phylogroups [49,50,51]. According to the results of the phylogenetic analysis, all Russian isolates, including those obtained in previous studies [21,22,52], with high bootstrap support belonged to phylogroups I and II (Supplementary Figure S3).

#### 3.2.2. Family: *Secoviridae*

##### Grapevine Fanleaf Virus

GFLV (genus *Nepovirus*) is one of the most harmful viral pathogens of grapevine [23,53]. It is responsible for fanleaf degeneration disease, the losses from which can reach up to 80% of the crop [54]. The severity of the disease and the intensity of the plant’s immune response vary depending on the strain [55]. There is no successful strategy to combat fanleaf degeneration [56], but a search for genetic factors of resistance is underway [57]. The presence of GFLV was confirmed using PCR in one sample (Supplementary Table S7). Satellite RNA was not detected. One complete genome was obtained using read mapping to the closest isolate (Supplementary Table S8).

GFLV has a bipartite +ssRNA genome. Phylogenetic analysis was performed for the complete sequences of RNA1 and RNA2 (Supplementary Figures S4 and S5). The novel Russian isolate on both dendrograms clustered close to the Russian isolates obtained during the study of the virome of the Dagestan ampelographic collections [22].

#### 3.2.3. Family: Betaflexiviridae

##### Grapevine Pinot Gris Virus

In 2012, the grapevine Pinot gris virus (GPGV) was discovered in vineyards in Italy; it was assigned to the genus *Trichovirus* [58]. Since then, this virus has been reported in more than forty countries around the world, including Russia [52]. GPGV is associated with grapevine leaf mottling and deformation (GLMD), but the virus is also detected in asymptomatic vines [59,60]. To date, there is no clear evidence regarding the relationship between GLMD symptoms and genetic variants of GPGV [61].

In this study, GPGV was identified and validated in 49 samples (Supplementary Table S7). By mapping reads to the closest genomes, 28 complete or nearly complete GPGV genomes were obtained (Supplementary Table S8). In the dendrogram, in most cases, the isolates obtained in this study clustered together with other Russian isolates (Supplementary Figure S6).

##### Grapevine Virus A

GVA (genus *Vitivirus*) is widely distributed throughout the world. This virus is involved in the development of rugose wood disease [32]. GVA has been associated with the Shiraz disease on susceptible red varieties, which causes primary bud necrosis, restricted spring growth, and decreased sugar accumulation in the berries [62,63,64,65]. This study identified GVA in 12 samples (Supplementary Table S7). Because GVA contigs were less than 90% identical to GVA isolates from the GenBank, the reads mapped poorly to reference genomes. Therefore, to assemble complete GVA genomes, mapping to whole-genome contigs was performed (Supplementary Table S8).

GVA genetic variants are divided into three molecular groups. Molecular group II isolates are associated with the Shiraz disease (SD) in South Africa [64] and Australia [63], but some variants within this group have been isolated from asymptomatic plants [65]. Group III variants are typically present in SD-susceptible plants that do not manifest symptoms of the disease [65]. Molecular group I is not believed to be associated with SD [63]. A phylogenetic analysis showed that the sequences clustered into three molecular groups (Supplementary Figure S7); representative isolates of each group were taken from the works of D. E. Goszczynski and O. J. Alabi [66,67]. Out of the genomes obtained in this study, two belonged to phylogroup I and clustered together with other isolates from Russia; 1 genome also belonged to phylogroup I but formed the same clade with isolates AF007415 and AY244516 which were allocated by O. J. Alabi et al. to additional phylogroup IV [67]. The fourth isolate obtained in this study belonged to phylogroup III. None of the Russian isolates belonged to phylogroup II, which is believed to be associated with the symptoms of Shiraz disease.

##### Grapevine Virus B

GVB (genus *Vitivirus*) belongs to pathogens of rugose wood [32]. In the LN33 grapevine hybrid, this virus can cause leaf reddening and longitudinally split cane wood [68]. In a study by Chitarra et al., infection of grapevines with GVB did not lead to economically significant consequences [69]. GVB was identified in two libraries (Supplementary Table S7). Mapping of reads to the closest genomes allowed us to obtain complete sequences of both isolates (Supplementary Table S8).

The existing genetic diversity of GVB is yet poorly understood. Most studies are based on comparisons of the CP gene [68,70,71], and whole-genome sequences are studied less often [72]. The phylogenetic analysis showed that the global GVB population is more genetically heterogeneous than previously thought (Supplementary Figure S8). The isolates were distributed into two large clusters, within which 3–5 phylogroups can be distinguished. The isolates obtained in this study belonged to the same cluster but to different phylogroups. The previously obtained genome from Russia [21] belonged to a different cluster.

##### Grapevine Virus F

GVF (genus *Vitivirus*) was first described in 2012 [73]. GVF was identified in one sample (Supplementary Table S7). By mapping reads to the reference sequence, a nearly complete genome of this isolate was obtained. A phylogenetic analysis showed that the isolates were divided into three molecular groups (Supplementary Figure S9). The GVF genome from this study was in the same clade as the Russian isolate obtained in the previous study [21] and three isolates from South Africa.

##### Grapevine Virus T

GVT (genus *Foveavirus*) was discovered in 2017 during a study of the grapevine transcriptome [74]. After PCR validation, GVT was identified in 15 samples (Supplementary Table S7). Using read mapping to the closest genomes (Supplementary Table S8), two nearly complete genomes were assembled. According to the literature, global GVT isolates are divided into seven molecular groups [75,76]. In a study of the ampelographic collection of the Republic of Dagestan of the Russian Federation, molecular group VIII of GVT was discovered [22]. The isolates from this study clustered with previously identified Russian isolates and belonged to group VIII (Supplementary Figure S10).

##### Grapevine Rupestris Stem Pitting-Associated Virus

GRSPaV (genus *Foveavirus*) is one of the most widespread grapevine viruses [77,78,79], including in Russia [80]. It is believed to be associated with rugose wood complex (RW) and rupestris stem pitting (RSP), but there is evidence that, in most cases, GRSPaV infection is asymptomatic [78]. GRSPaV was identified in 50 libraries (Supplementary Table S7). Complete or nearly complete genomes were obtained for 41 isolates.

Molecular groups of GRSPaV are identified both on the basis of CP and RdRp sequences [78,79,81,82,83] and on the basis of complete genomes [78,82]. In both cases, it is customary to distinguish phylogroups 1, 2a, 2b, 2c, 3, and 4 that are divided into subgroups. The phylogenetic analysis was performed using complete nucleotide sequences of GRSPaV genomes. The last study of GRSPaV genetic diversity based on complete genomes was conducted in 2018 [78]. For the phylogenetic analysis of Russian GRSPaV isolates, we took 41 genomes assembled in this study, 18 genomes of representative members of phylogroups [78,82], all Russian GRSPaV isolates from the GenBank (44 genomes), and 53 representative genomes of phylogroups of isolates published in the GenBank since 2018. The GRSPaV genomes obtained in this study belonged to all six phylogroups, with 27 genomes (66%) belonging to group 1, as well as most other Russian isolates (Supplementary Figure S11). Within group 1, our isolates with high bootstrap support were divided into at least four more subgroups. Nine genomes belonged to group 3 (22%), two genomes belonged to group 2a, and three genomes belonged to the remaining three groups (1 genome per group).

#### 3.2.4. Family: *Tymoviridae*

##### Grapevine Fleck Virus

GFkV (genus *Maculavirus*) is distributed worldwide, it is often present in a latent state in grapevines [32]. It may cause localized translucent spots on young leaves due to clearing of the veins of the third and fourth order, especially on *Vitis rupestris* [84]. GFkV was identified in 41 samples (Supplementary Table S7). Complete genomes were not assembled.

##### Grapevine Red Globe Virus

GRGV (genus *Maculavirus*) is poorly described in the literature. It was registered on grapevines on all continents [32,85,86]. Probably, GRGV does not cause any noticeable symptoms [84,86]. GRGV was identified in three samples (Supplementary Table S7). Due to low coverage, complete genomes were not assembled. 

##### Grapevine Syrah Virus 1

GSyV-1 (genus *Marafivirus*) was discovered in 2009 in the USA [87]. Despite the widespread occurrence of this virus in vineyards worldwide, there is no information on the diseases it causes [84]. In this study, GSyV-1 was identified in 29 samples (Supplementary Table S7). By mapping reads to the reference sequence, one nearly complete genome was assembled.

In 2015, a study of molecular diversity among GSyV-1 isolates in the CP gene sequences showed the division of isolates into two groups, the first of which consisted of two subgroups [88]. In this study, the whole-genome sequences of GSyV-1 were also divided into two groups, with three subgroups allocated to the first group (Supplementary Figure S12). Thus, four molecular groups of GSyV-1 were identified. The isolate assembled in this study belonged to the second group.

##### Grapevine Asteroid Mosaic-Associated Virus

GAMaV (genus *Marafivirus*), together with several related viruses, constitutes the so-called fleck complex [32]. Infection caused by GAMaV is characterized by star-shaped chlorotic spots on leaves and may be latent [84]. This virus was found not only in production vineyards and collections [21] but also in free-living grapevines [89]. GAMaV was identified in four libraries (Supplementary Table S7). Due to low coverage, complete genomes were not assembled.

##### Grapevine Rupestris Vein Feathering Virus

GRVFV (genus *Marafivirus*) is also a part of the fleck complex [32]. The infection is often latent, with symptoms similar to GFkV and GAMaV. On *V. rupestris*, the GRVFV infection manifests itself as a transient chlorotic feathering of the primary and secondary veins [84]. GRVFV was identified in 30 libraries (Supplementary Table S7). To assemble complete genomes, reads were mapped with low sensitivity (10% mismatches per read) to the closest genomes (Supplementary Table S8). As a result, four genomes were obtained.

To date, no studies have been conducted on the molecular diversity of GRVFV. In this study, world isolates showed the existence of at least four molecular groups; genomes obtained in this study belonged to three different groups (Supplementary Figure S13). No relationship was found between phylogroups and the country of origin of the viruses. 

##### Grapevine-Associated Tymo-like Virus

GaTLV was first described in 2018 in France and tentatively assigned to a new genus, *Gratylivirus,* in the family *Tymoviridae* [90]. Then, this virus was detected in the USA [5,91] and Canada [92]. In Russia, GaTLV was discovered for the first time in this study. The symptoms associated with GaTLV are currently unknown [3]. Moreover, the detection of GaTLV on infected grapevines appears to be very unstable throughout the growing season, suggesting that the host of this virus may be a grapevine-associated organism [90]. In this study, GaTLV was found in 40 samples (Supplementary Table S7). By mapping reads to the reference genome NC_040837, 13 complete and nearly complete genomes were obtained.

A phylogenetic analysis showed that isolates from this study clustered together; the reference isolate from France was in the same group (Supplementary Figure S14). Three isolates from the USA formed a separate cluster.

#### 3.2.5. Family: *Partitiviridae*

##### Vitis Cryptic Virus

VCV was discovered in 2021 on a wild *Vitis coignetiae* in northern Japan [93]. It is a dsRNA virus from the family *Partitiviridae* with two segments encoding the RdRp and CP genes, respectively. It was found in cultivated grapevines in China [94] and Russia [21]. To date, the symptoms associated with VCV are unknown. VCV was identified in two libraries, and the complete sequences of both RNAs were obtained for one sample. At the time of this study, only six complete VCV genomes were available in the world: two from Russia and four from Japan. A phylogenetic analysis (Supplementary Figure S15) showed low values of bootstrap support of nodes (45–70). It is possible that more genome sequences need to be accumulated to determine the phylogenetic relationships of VCV.

#### 3.2.6. Family: *Tombusviridae*

##### Grapevine Umbra-like Virus

When studying the virome of ampelographic collections of Anapa and Dagestan in 2022, a novel grapevine umbra-like virus (GULV) from the family *Tombusviridae* was discovered [21,22]. This study confirms the presence of this virus in grapevines from the Don ampelographic collection. GULV was identified in 10 samples (Supplementary Table S7). By mapping reads to isolate OP886321, six whole-genome GULV sequences were obtained. A phylogenetic analysis showed that the isolates obtained in this study are similar to each other and to those detected earlier (Supplementary Figure S16).

#### 3.2.7. Family: *Caulimoviridae*

##### Grapevine Pararetrovirus

GPRV was discovered in 2022 during a virome study of ampelographic collections of Dagestan, Russia [22]. It has a circular dsDNA genome. In this study, GPRV was identified in 19 samples (Supplementary Table S7).

#### 3.2.8. Family: Unassigned

##### Grapevine Satellite Virus

GV-Sat (genus *Virtovirus*) was discovered in 2013 in the USA [95], then it was detected on grapevines from Iran [96], Hungary [97], Slovenia [98], and Russia [21]. GV-Sat was identified in three samples (Supplementary Table S7), but the number of reads was too small to assemble whole-genome sequences. The helper virus for GV-Sat is unknown. In this study, GV-Sat co-infected plants along with many viral pathogens. Analysis of obtained data confirmed by literature sources shows that GLRaV-1 is always detected together with GV-Sat (Supplementary Table S9), which may indicate its role as a helper in relation to GV-Sat [22,98].

#### 3.2.9. Family: *Pospiviroidae*

##### Hop Stunt Viroid

HSVd (genus *Hostuviroid*) is ubiquitous and can infect citrus and stone fruit trees, as well as hops and grapevines [99,100]. Most plants, including grapevine, typically contain HSVd in a latent form [101]. In Japan, grapevines served as an asymptomatic reservoir of HSVd, through which the infection was transmitted to hop crops and led to a hop stunt epidemic [102]. HSVd was identified in 50 samples (Supplementary Table S7). As a result of a contig analysis, 68 complete HSVd sequences were obtained. For 16 libraries, more than one genome was assembled. Some genomes obtained in different libraries showed 100% identity with each other. This was taken into account when conducting phylogenetic analysis; however, only one copy of each genetic variant was submitted to GenBank. HSVd phylogroups were determined based on the study by Maddahian et al. [99]. The HSVd genomes obtained in this study belonged to three phylogroups: 7 to the citrus group, 15 to the plum-hop/cit3 group, and 46 to the hop group (Supplementary Figure S17).

##### Grapevine Yellow Speckle Viroid 1

GYSVd-1 (genus *Apscaviroid*) is the most widespread pathogen of grapevine yellow speckle disease [103]. In most cases, the infection is latent; yellow spots scattered on the leaf blade or gathering along the main veins are observed in susceptible plants [104]. One of the factors that contribute to the development of symptoms is a warm climate [103]. GYSVd-1 was identified in 48 samples (Supplementary Table S7). Contig analysis allowed us to assemble 48 complete GYSVd-1 sequences, with nine libraries yielding two genomes each. As in the case of HSVd, 100% identical genomes from different libraries were included in the phylogenetic analysis but submitted to GenBank in a single copy.

There are four known molecular types for GYSVd-1 [105,106]. In this study, type 2 and type 3 were identified on the dendrogram, but sequences belonging to type 1 and type 4 formed one cluster (Supplementary Figure S18). Russian isolates were distributed throughout the dendrogram: type 2 and type 3 comprised 11 genomes each; the remaining 26 genomes were grouped with isolates from all over the world. Thus, the data obtained indicate a large genetic diversity of GYSVd-1 in Russia. 

##### Grapevine Yellow Speckle Viroid 2

GYSVd-2 (genus *Apscaviroid*) is a less common pathogen of grapevine yellow speckle disease [103]. GYSVd-2 was identified in 14 samples (Supplementary Table S7). Using the contig analysis, 15 complete GYSVd-2 genomes were assembled, with the S1798 library yielding two genomes. As in the case of HSVd and GYSVd-1, 100% identical genomes were submitted to the GenBank in a single copy, but all genomes participated in the phylogenetic analysis. On the dendrogram, 60% of the genomes obtained in this study were grouped with isolates from Asia (Iran, China, India, Thailand, and the Republic of Korea), 20% formed a separate cluster, the remaining 20% were part of a mixed group with isolates from China, South Africa, Russia, Italy, Greece, Chile, Pakistan, and Iran (Supplementary Figure S19). 

##### Australian Grapevine Viroid

AGVd (genus *Apscaviroid*) was first discovered in Australia [107] but has now been found to have a limited distribution worldwide [103]. AGVd was identified in 14 samples (Supplementary Table S7). Complete sequences were obtained for seven isolates. AGVd sequences are known to be characterized by low molecular diversity [104], which is supported by the data presented here. Russian isolates, including those identified earlier, were distributed throughout the dendrogram and were grouped with low bootstrap support values together with isolates from different geographical locations (Supplementary Figure S20).

### 3.3. Detection of Novel Grapevine Viruses

#### Grapevine Alphapartitivirus (GAPV)

Using the SPAdes assembler, a 731 bp contig was *de novo* assembled from the S1773 library. A tblastx analysis showed its identity with the beet cryptic virus 1 RNA1 (NC_011556.1), E-value 1.09 × 10^−122^. The megablast algorithm of blastn did not show any identity between the detected nucleotide sequence and those available in the GenBank database, which allowed us to assume the presence of a novel virus in the sample.

Beet cryptic virus 1 belongs to the family *Partitiviridae* with a linear two-segmented dsRNA genome. To search for other contigs, we produced a database of all 3575 nucleotide sequences of viruses of the family *Partitiviridae* available in the GenBank. Next, for contigs from the S1773 library assembled using the Geneious and SPAdes assemblers, a tblastx analysis was performed against this database. Contigs that belonged to plant partitiviruses were compared with the GenBank database using blastn (megablast, discontiguous megablast), blastx, and tblastx. As a result, two more contigs for RNA1 (312 bp and 287 bp) and one contig for RNA2 (839 bp) were found. The mapping of reads from all libraries was performed to all four contigs with several iterations, which made it possible to extend the RNA1 contigs to 862 bp, 422 bp, 390 bp, and RNA2 to 1045 bp.

At the next stage, to assemble the complete genome, the resulting RNA1 contigs were translated and aligned with the translated sequence of the closest beet cryptic virus 1 (NC_011556.1). Amino acid sequence identity was 64.6%, 71.5%, and 72% for each contig. Specific primers were designed to close gaps in the nucleotide sequence, and the resulting PCR products were Sanger-sequenced. As a result of the assembly of the whole-genome sequence and amplification of the 5′ and 3′ terminal regions, complete RNA1 1980 nt in length and RNA2 1710 nt in length were obtained for the isolate from the library S1807.

**Figure 1:**
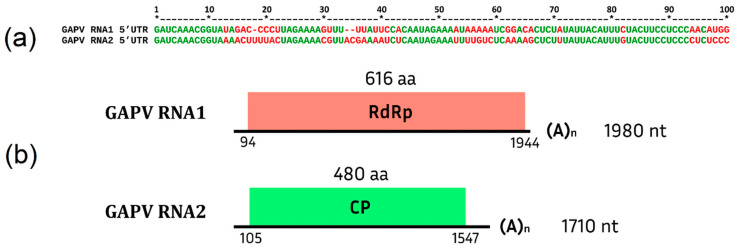
(**a**) Conserved sequences of the 5′ terminal regions of GAPV RNA1 and RNA2; (**b**) genome organization of GAPV. Each genomic segment contains one ORF.

Partitiviruses are known to be characterized by conserved 5′ regions on the plus strands of RNA1 and RNA2 [108,109]. Alphapartitiviruses, to which beet cryptic virus 1 belongs, have a genus-specific sequence GAWNW at the 5′ end [110]. The alignment of RNA1 and RNA2 showed a high level of identity in the first 100 nucleotides of the 5′ end of RNA1 and RNA2 and the presence of a genus-specific GAWNW region (Figure 1a), which indicates that both RNAs belong to the same virus species that was tentatively named grapevine alphapartitivirus (GAPV). Using RT-PCR with primers specific to RNA1 and RNA2, both RNAs were detected in seven libraries (Supplementary Table S7).

GAPV has a genome organization typical for representatives of this family (Figure 1b). RNA1 contains one ORF 1851 nt in length encoding an RNA-dependent RNA polymerase, and RNA2 contains one ORF 1443 nt in length. The function of the protein encoded in it was not predicted by InterPro, but as a result of a blastp analysis using the GenBank database, similarities with the coat protein of partitiviruses were discovered.

**Figure 2:**
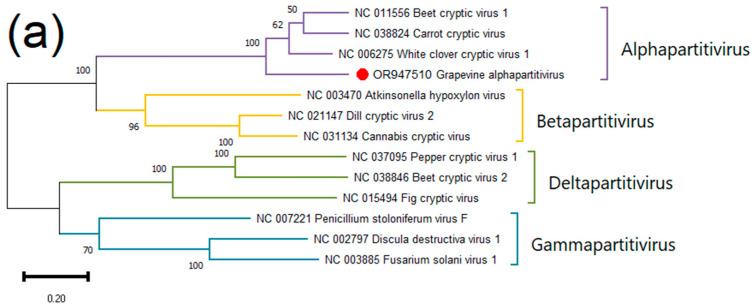

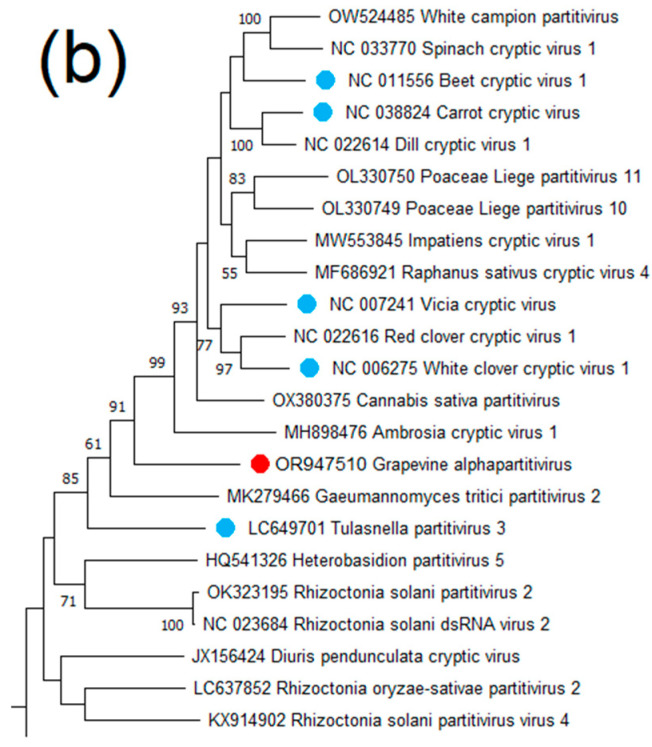
(**a**) Phylogenetic tree based on nucleotide sequences of RNA1 of GAPV (red dot) and representative members of the family *Partitiviridae*; (**b**) Fragment of phylogenetic tree based on nucleotide sequences of RNA1 of GAPV (red dot), alphapartitiviruses (blue dots), and unclassified partitiviruses. The full tree is contained in Supplementary Figure S21. The trees were constructed in MEGA11 using the maximum-likelihood method and GTR model with 1000 bootstrap replicates. Bootstrap values below 50 are not shown.

To determine the position of GAPV among the partitiviruses, a phylogenetic tree was constructed based on the nucleotide sequences of GAPV RNA1 and representative members of genera from the family *Partitiviridae*. In the dendrogram, GAPV clustered together with alphapartitiviruses (Figure 2a). For a more detailed phylogenetic analysis, we took 28 complete nucleotide sequences of RNA1 of alphapartitiviruses and 41 sequences of unclassified partitiviruses that showed >40% nucleotide sequence identity with GAPV. The closest neighbors of GAPV included both plant and fungal partitiviruses (Figure 2b).

**Figure 3:**
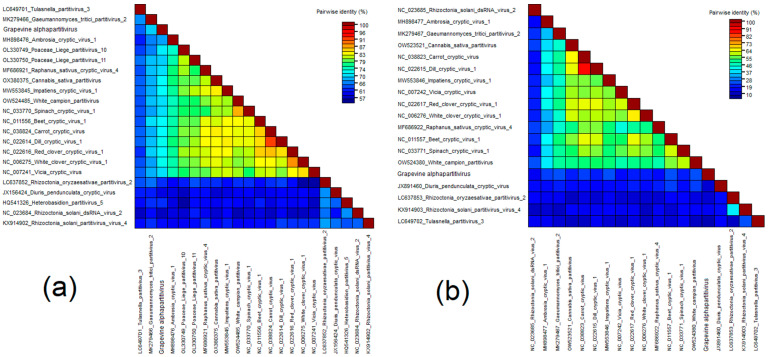
Pairwise identity matrix including amino acid sequences of (**a**) RdRp and (**b**) CP of GAPV and closest partitiviruses.

The amino acid sequences of viral RdRp and CP genes clustered on the dendrogram next to GAPV were used for SDT analysis. As a result, the RdRp identity for the partitivirus discovered with the closest species was ≤72.1%, while the CP identity was ≤23.6% (Figure 3 and Supplementary Tables S10 and S11). The demarcation criteria for a species in the genus *Alphapartitivirus* are ≤90% aa sequence identity in the RdRp and ≤80% aa sequence identity in the CP [111]. Thus, GAPV can be considered a novel species belonging to the genus *Alphapartitivirus*.

During bioinformatics analysis, the reads belonging to GAPV were noted in 16 libraries. At the same time, an extremely low coverage was observed: from 1 to 47 reads per library. To successfully validate GAPV using RT-PCR, the number of PCR cycles had to be increased from 35 to 40. As a result, GAPV was validated in 23 libraries. In 11 of them, not a single read of this virus was detected. This may be due to the low titer of GAPV in a plant or the difficulty of its identification using the HTS method.

The genus *Alphapartitivirus* includes plant and fungal viruses [111]. On the dendrogram, GAPV is in close proximity to viruses associated both with plants and with fungi. The results of the SDT analysis indicate that the amino acid sequence of RdRp GAPV is closer to plant viruses. However, the close phylogenetic relationship of plant and fungal alphapartitiviruses requires attentiveness when assigning them to one or another host, especially considering that Nerva et al. (2017) [112] have previously demonstrated that a mycovirus from the family *Partitiviridae* is able to successfully replicate in plant cells without changes in the nucleotide sequence. Thus, identifying the host for GAPV is challenging and requires additional research.

Due to the multiple infections of the grapevines on which GAPV was detected, the presence of the novel virus cannot be associated with any particular symptoms. Partitiviruses are generally associated with persistent infections, and the only known way they spread is through seeds [111]. Interestingly, some of the partitiviruses have a mutualistic relationship with their host plants [113,114]. This may be characteristic of many plant partitiviruses [110,115,116] and is therefore of interest to study in relation to GAPV.

#### Grapevine Secovirus (GSV)

Using the SPAdes assembler, a contig 594 bp in length was assembled from the S1823 library; a tblastx analysis showed its identity with RNA1 of the Prunus virus F (E-value 3.50 × 10^−47^), which has a two-segmented (+) RNA genome and belongs to the order *Picornavirales*, family *Secoviridae*, subfamily *Comovirinae*, genus *Fabavirus*. A blastn analysis of this contig against the GenBank nucleotide sequence database with default parameters found no similarities to known organisms.

We produced a database of 5570 nucleotide sequences of the order *Picornavirales* from the GenBank; human, mammalian, and avian viruses were excluded. As a result of tblastx analysis of contigs from the S1823 library against this database, six more contigs presumably belonging to a novel virus were discovered. These contigs did not produce matches when a blastn analysis was performed against the GenBank database, but a blastx analysis showed that they have similarities to viruses from the *Secoviridae* family. The closest was the yucca gloriosa secovirus (unclassified *Secoviridae*, tentatively assigned to *Fabavirus*), discovered in 2022 during an analysis of the grape transcriptome [117]. At the same time, six contigs of various lengths matched RNA1, and one contig of 3546 bp in length matched RNA2.

The identified contigs were translated and aligned with the translated genome of yucca gloriosa secovirus (RNA1 BK061335.1 and RNA2 BK061336.1), which made it possible to assemble out of these six contigs, two contigs 3260 bp and 1347 bp in length which corresponded to RNA1. The gap between contigs was amplified using specific primers and Sanger-sequenced. As a result of amplification of the 5′ and 3′ terminal regions, the complete genome sequence of a virus tentatively named grapevine secovirus (GSV) was obtained, with a length of 6290 nt for RNA1 and 5941 nt for RNA2. Using RT-PCR with RNA1- and RNA2-specific primers, GSV was confirmed in one library (Supplementary Table S7).

**Figure 4:**
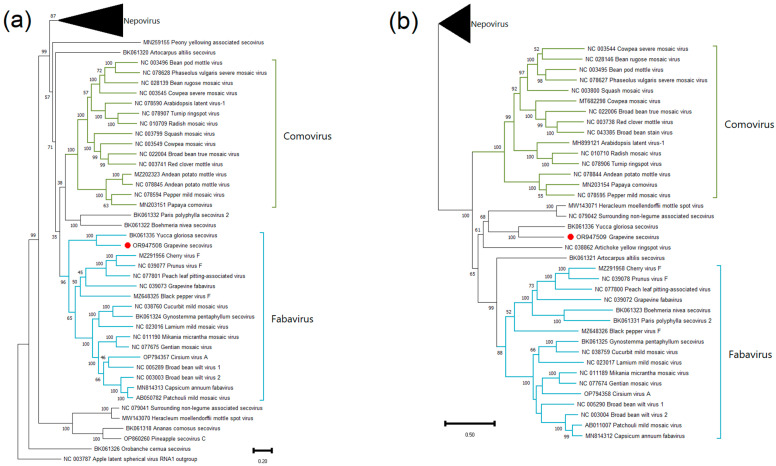
Phylogenetic trees based on nucleotide sequences of (**a**) RNA1 and (**b**) RNA2 of GSV (red dot) and members of the genera *Nepovirus*, *Fabavirus*, *Comovirus*, and unclassified *Secoviridae*. The trees were constructed in MEGA11 using the maximum-likelihood method and GTR model with 1000 bootstrap replicates.

For phylogenetic analysis, nucleotide sequences of RNA1 and RNA2 of GSV and viruses of the genera *Nepovirus, Fabavirus, Comovirus,* and unclassified *Secoviridae* were used. On dendrograms, GSV clustered together with fabaviruses in a separate clade with yucca gloriosa secovirus (Figure 4).

A distinctive feature of GSV is the large genome length of RNA1 (6290 nt) and RNA2 (5941 nt) compared to 5800–6000 nt and 3300–4000 nt for RNA1 and RNA2 of other fabaviruses, respectively [118]. At the same time, an isolate of yucca gloriosa secovirus that is phylogenetically close to GSV has a genome of comparable size (6309 and 5477 nt for RNA1 and RNA2, respectively).

**Figure 5:**
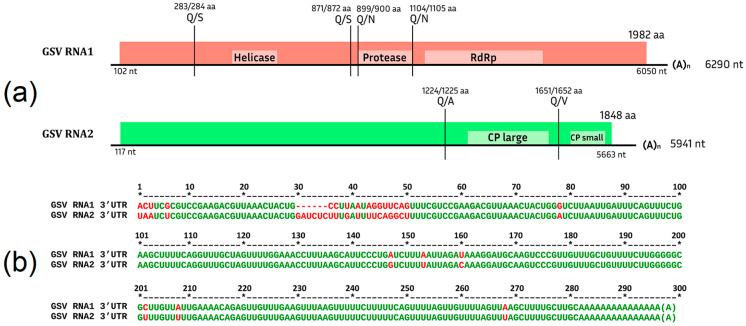
(**a**) Putative genome organization of GSV. Cleavage sites were predicted by multiple alignments of the aa sequences with fabaviruses. Light colors indicate conserved domains predicted by InterPro; (**b**) conserved sequences of the 3′ terminal regions of GSV RNA1 and RNA2.

The organization of the GSV genome is typical for fabaviruses (Figure 5a). The 3′-UTR regions of RNA1 and RNA2 have a conservative structure (Figure 5b). RNA1 contains one ORF encoding a polyprotein 1982 aa in length with conservative domains of type III helicase, 3C-like cysteine proteinase, and type I polymerase. As a result of multiple alignments with sequences of fabaviruses, proteolytic cleavage sites were predicted. RNA2 contains an ORF encoding a polyprotein 1848 aa in length with conservative domains of large and small coat proteins characteristic of the genera *Fabavirus* and *Comovirus*. The size of the GSV N-terminal polyprotein is 1224 aa, approximately two times larger than in other representatives of the genera *Fabavirus* and *Comovirus*. The function of this polyprotein could not be predicted using InterPro.

For phylogenetic analysis of the amino acid sequences of the Pro-Pol region of RNA1 and the coat protein of RNA2, we used all representatives of the genera *Nepovirus, Fabavirus* and *Comovirus*, as well as unclassified *Secoviridae*. On both dendrograms, GSV clustered next to yucca gloriosa secovirus and belonged to the fabavirus clade (Supplementary Figures S22 and S23).

**Figure 6:**
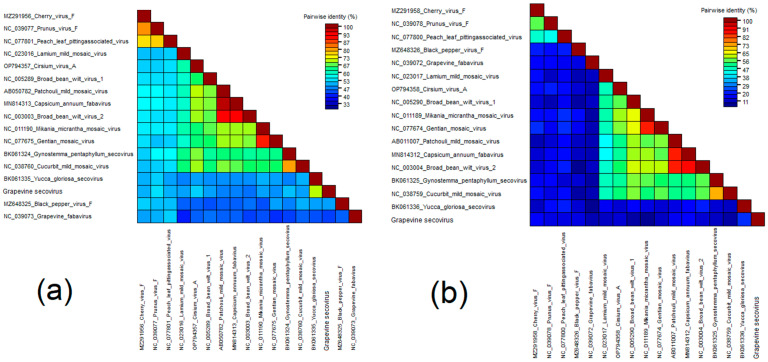
Pairwise identity matrix including amino acid sequences of the (**a**) Pro-Pol region and (**b**) CP of GSV and fabaviruses.

The Pro-Pol and CP amino acid sequences of viruses that were clustering on dendrograms with fabaviruses were used for the SDT analysis. As a result, the Pro-Pol and CP sequences GSV were 69.9% and 22.5% identical to sequences belonging to the yucca gloriosa secovirus (Figure 6 and Supplementary Tables S12 and S13). For the *Secoviridae* family, the following species demarcation criteria were applied: conserved Pro-Pol region aa sequence with less than 80% identity and CP aa sequence with less than 75% identity [118]. Thus, GSV can be considered a novel species belonging to the genus *Fabavirus*.

The family *Secoviridae* includes many economically important plant pathogens [119]. Secoviruses can cause the following symptoms: ringspots, mottling, mosaic, distortion, wilting, and apical necrosis [118]. In this study, GSV was detected in one plant. This grapevine was infected with four viruses, including two described for the first time in this study. Therefore, there is not yet enough data to associate specific symptoms with the presence of GSV in the plant.

Like members of the phylogenetically related genera *Nepovirus, Fabavirus,* and *Comovirus* [118,120,121], GSV has a bipartite (+) ssRNA genome. Each genomic RNA encodes a large polyprotein, which is cleaved by 3C-like proteinases. The protein domains predicted for GSV RNA1 are typical of the entire family *Secoviridae* [120]. When analyzing the GSV genome, it was established that the RNA2 polyprotein contains large and small capsid proteins, like comoviruses and fabaviruses. Cleavage sites were predicted based on the assumption that GSV belongs to fabaviruses, as described previously [122,123,124,125,126]. However, the amino acid sequence of the N-terminus prior to the beginning of the large CP is much longer than that in fabaviruses and does not align with the N-terminus of the fabavirus RNA2 polyprotein. Therefore, it is likely that there may be other cleavage sites in this region of the GSV RNA2 polypeptide.

The presence of RNA2, which is unusually long for fabaviruses, and the phylogenetic distance of both GSV RNAs from other fabaviruses raises the question of whether this virus, together with the similar yucca gloriosa secovirus, are representatives of a new genus of the family *Secoviridae*. To clarify their taxonomic position, it is necessary to accumulate more knowledge about the structure, organization, and expression of the genome, as well as the biology of these viruses.

#### Grapevine Umbra-like Viruses

A tblastx analysis of contigs showed their identity with umbraviruses in 10 libraries; however, a blastn analysis in the megablast mode did not detect matches between these contigs and sequences from the NCBI GenBank. This allowed us to assume the presence of new umbra-like viruses in the samples.

In nine libraries, these contigs, approximately 3540 bp in length, showed identity with Patrinia mild mottle virus (NC_055564.1) with E-values ranging from 3.28 × 10^−86^ to 1.56e-105. As a result of multiple alignments, it was found that they could be divided into two groups that were less than 36.8% identical to each other. The identity within each group was >99%.

Contigs of the first group are present in libraries S1774, S1797, S1801, S1820. From these contigs, we assembled a consensus nucleotide sequence 3404 bp in length, which corresponded to the nearly complete genome of umbraviruses. Mapping the reads from each of these libraries to the resulting sequence allowed us to assemble complete or nearly complete genomes of four isolates. Their identity with each other was 99.2–99.4%. As a result of the amplification of the 5′ and 3′ terminal regions, the complete sequence of an isolate from the S1820 library with a length of 3410 nt was obtained.

Contigs of the second group are present in libraries S1745, S1749, S1752, S1823, and S1824. The scheme of their analysis was the same as for the contigs of the first group. As a result, a consensus sequence of 3555 bp in length was obtained. After mapping the reads from these libraries, complete or nearly complete nucleotide sequences of five isolates were obtained. Their identity was 97.6–98%. As a result of the amplification of the 5′ and 3′ terminal regions, the complete nucleotide sequence of an isolate from the S1823 library with a length of 3563 nt was obtained.

In the tenth library (S1770), as a result of tblastx analysis, three contigs were identified with a length of 1780 bp, 2778 bp, and 2782 bp that had an identity with Carrot mottle virus (NC_011515.1) with an E-value ranging from 1.45 × 10^−87^ to 6.77 × 10^−115^. The two long contigs were 98.17% identical, with all differences occurring at the 5′ end. Their presence in the sample was confirmed using RT-PCR with specific primers. For the 2782 nt contig, the 5′ and 3′ terminal regions were sequenced. As a result, the length of the complete nucleotide sequence comprised 3764 nt. The third contig from the S1770 library, 1780 nt in length, had an 86.91% identity with the other two contigs. It was not possible to obtain a sequence corresponding to the complete genome of this isolate, but its presence was confirmed by RT-PCR using specific primers.

A pairwise comparison of three whole-genome sequences from libraries S1770, S1820, and S1823 confirmed that they belong to genomes of different viruses. These sequences were tentatively named grapevine umbra-like virus 2 (GULV-2), GULV-3, and GULV-4 (Table 1). Validation using RT-PCR with specific primers confirmed their presence in 17 samples (Supplementary Table S7).

**Figure 7:**
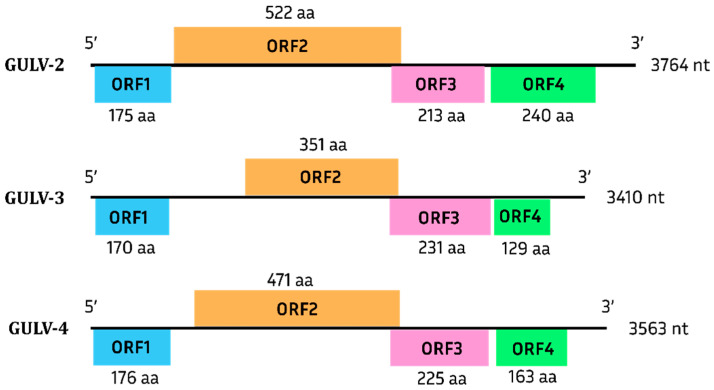
Putative genome organization of GULV-2, -3, -4. ORF1 and ORF2 encode RdRp, ORF3 and ORF4 encode proteins with unknown function.

GULV-2, GULV-3, and GULV-4 have similar genome organization and contain 4 ORFs (Figure 7), with ORF2 and ORF3 partially overlapping. The products of ORF1 and ORF2 have similarities to the RdRp of representatives of the *Tombusviridae* family, while the products of ORF3 and ORF4 show no similarity to proteins from the databases.

**Figure 8:**
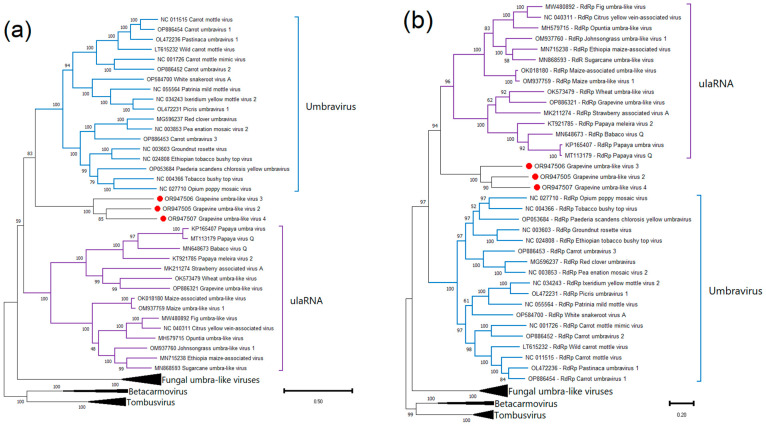
Phylogenetic trees based on nucleotide sequences of (**a**) genomes and (**b**) RdRp of GULVs (red dots), umbraviruses, and ulaRNAs. The tree was constructed in MEGA11 using the maximum-likelihood method and GTR model with 1000 bootstrap replicates.

Phylogenetic analysis was carried out using nucleotide sequences of complete genomes and RdRp (ORF1 + ORF2) of all umbraviruses and umbra-like viruses available in the GenBank. As a result, on both dendrograms, all fungal umbra-like viruses clustered separately from plant umbraviruses and umbra-like viruses (Figure 8). The GULV-2, -3, and -4 sequences clustered together and occupied an intermediate position between umbraviruses and plant umbra-like viruses (ulaRNAs). Previously discovered grapevine umbra-like virus clustered in the clade of plant umbra-like viruses along with wheat umbra-like virus and strawberry-associated virus A.

**Figure 9:**
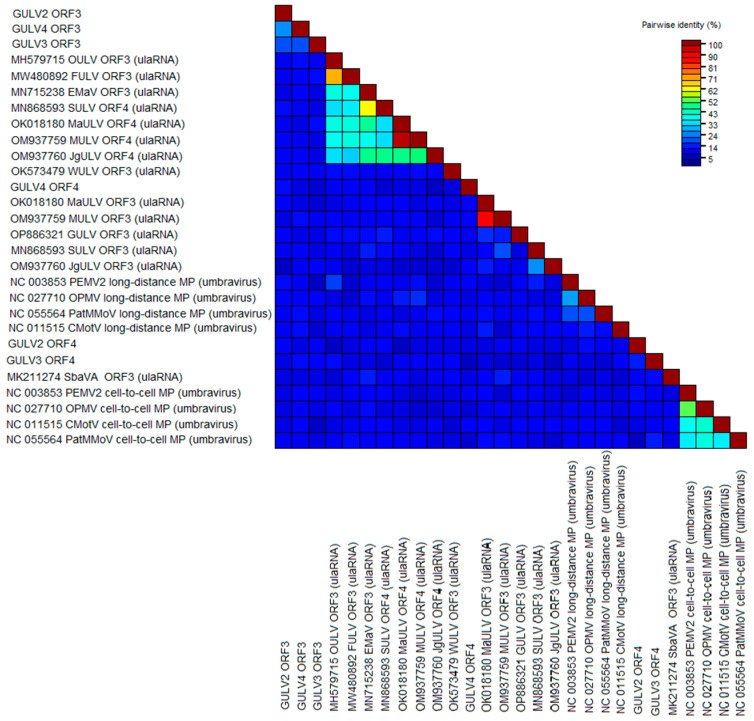
Pairwise identity matrix including amino acid sequences of ORF3 and ORF4 of GULVs, four umbraviruses, and ten ulaRNAs. OULV: opuntia umbra-like virus; FULV: fig umbra-like virus; EMaV: Ethiopia maize-associated virus; SULV: sugarcane umbra-like virus; MaULV: maize-associated umbra-like virus; MULV: maize umbra-like virus 1; GULV: grapevine umbra-like virus; JgULV: johnsongrass umbra-like virus 1; PEMV2: pea enation mosaic virus 2; OPMV: opium poppy mosaic virus; PatMMoV: patrinia mild mottle virus; CmotV: carrot mottle virus; SbaVA: strawberry associated virus A.

To check the similarity of the products of ORF3 and ORF4 of GULVs to the proteins of closest viruses, SDT analysis was performed with the amino acid sequences of ORF3 and ORF4 of four umbraviruses and ten ulaRNAs for which the corresponding ORFs are known (Figure 9 and Supplementary Table S14). As a result, ORF3 of GULV-2, -3, and -4 were 18.7–24.9% identical to each other. All other analyzed sequences showed less similarity to products of ORF3 and ORF4 of GULVs.

For the SDT analysis, the nucleotide sequences of the genomes and RdRp gene of GULV-2, -3, -4, all umbraviruses, and plant umbra-like viruses (ulaRNAs) available in the GenBank were used. The pairwise comparisons matrix showed that GULV-2, -3, and -4 have less than 50% identity with the closest known sequences (Table 2 and Supplementary Tables S15 and S16). The comparison results again indicate an intermediate position of GULV-2, -3, and -4 between umbraviruses and ulaRNAs.

The demarcation criterion for identifying a new species in the genus *Umbravirus* is the nucleotide sequence identity of less than 70% [127]; therefore, the GULV-2, -3, -4 discovered by us can be classified as three new species. We followed the traditional ICTV nomenclature and considered GULVs to be viruses, but their properties are similar to the recently described group of umbravirus-like associated RNAs (ulaRNAs) [128,129].

GULVs were moderately abundant in our samples: GULV-4 was detected in 20% of the examined plants, GULV-3 in 10%, and GULV-2 in 6%. At the same time, three samples were found to be co-infected with GULV-4 and either GULV-2 or GULV-3. Contig analysis showed that GULV-2 existed as a population in the S1770 library. We were able to validate all three variants of this virus using PCR, one of which should probably be considered a partial sequence of a separate species. All GULV-positive samples were susceptible to mixed viral infections; therefore, specific plant symptoms cannot be attributed to GULVs.

As is known, the genus *Umbravirus* includes viruses that do not encode coat proteins [130]. To spread between host plants, they require helper viruses from the family *Solemoviridae* [131]. Viruses of this family were not detected in our samples nor in other studies of plants with ulaRNAs [132,133,134,135].

The genome organization of GULVs is similar to ulaRNAs. ORF1 and ORF2 of typical umbraviruses and ulaRNAs encode RdRp due to −1 programmed ribosomal frameshifting (−1 PRF) or suppression of termination at a stop codon [136,137]. This is probably true for GULVs as well. Some ulaRNAs, like GULVs, contain ORF3 and ORF4; however, unlike umbraviruses, they do not overlap, and instead of long-distance movement proteins and cell-to-cell movement proteins, they encode unknown proteins [132,133,134,135]. Moreover, ORF2 of GULVs partially overlaps with ORF3, which is also uncharacteristic of umbraviruses but has been described for such ulaRNAs as fig umbra-like virus, opuntia umbra-like virus, strawberry-associated virus A, wheat umbra-like virus, and grapevine umbra-like virus [22,132,133]. Comparing the amino acid sequences of ORF3 and ORF4 showed that these GULV proteins are not similar to those of umbraviruses and ulaRNAs. 

In the phylogenetic analysis, GULVs form a distinct clade. On a dendrogram based on the nucleotide sequences of the RdRp gene, they cluster closer to ulaRNAs, while on a dendrogram based on the sequences of complete genomes, they cluster closer to umbraviruses. Thus, GULVs occupy an intermediate position between members of the *Umbravirus* genus and ulaRNAs.

### 3.4. Analysis of Mixed Infections

This study used two approaches to identify viruses and viroids: bioinformatics analysis of total RNA sequencing data and RT-PCR with primers specific to the genomes of viruses and viroids. In a number of cases, the results of detection by these methods did not match. The greatest number of discrepancies was found for (+) ssRNA GFkV, dsRNA GAPV, and dsDNA GPRV, for which more positive samples were identified using RT-PCR than using HTS. Samples with the number of reads at the threshold level were also difficult to analyze. This may be associated both with a low virus titer in the plant and with the presence of contamination during sequencing. To achieve a compromise between reduced sensitivity of bioinformatics analysis and false-positive identification, the samples confirmed by both methods were considered to be positive. The use of two methods complements each other and makes it possible to conclude whether a virus is present in a sample more accurately. Thus, of particular importance is a detailed analysis of data based on expert experience for each virus individually, taking into account the success of their sequencing and validation in each specific dataset.

**Figure 10:**
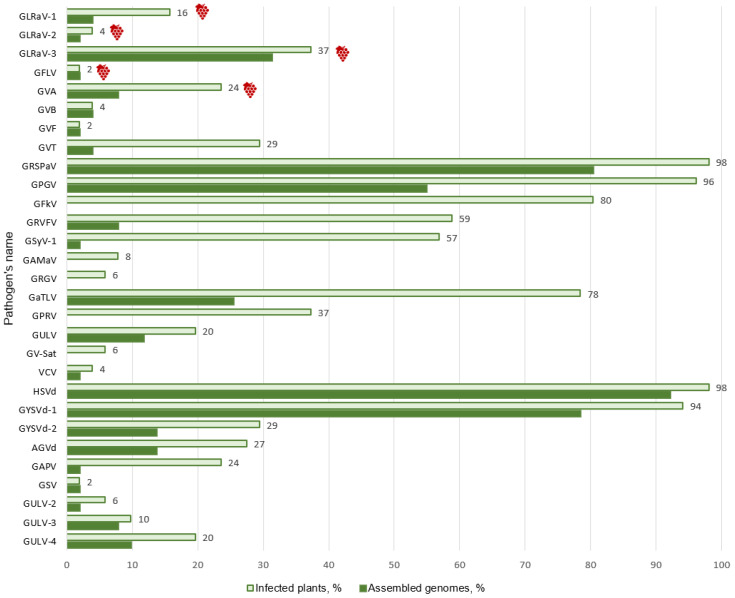
Frequency of spread of grapevine viruses and viroids in the Don ampelographic collection (percentage of the total number of collected samples). Economically significant viruses are shown as 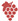.

As a result of the analysis of 51 grapevines with symptoms of viral infection from the Don ampelographic collection (Russia), 20 previously described viruses and 4 viroids were identified, along with five novel viruses (Figure 10). All plants were infected with 3 to 16 viruses and viroids (Supplementary Table S1). The least infected sample was S1740 of the Russian cultivar Tsimlyansky Black with GRSPaV, GRFVF, and HSVd. The most infected sample was S1797 of the Moldovan cultivar Plavay, with 16 viruses and viroids.

In general, the most widespread viruses on the grapevine of the collection were GRSPaV, GPGV, and GFkV. They were found in 80-98% of the samples. Most samples were infected with HSVd and GYSVd-1—98% and 94%, respectively. The proportions of the other two viroids detected, GYSVd-2 and AGVd, were 29% and 27%, respectively. Among the economically significant pathogens of grapevine, the most widespread was GLRaV-3, which affected 19 analyzed plants (37%). At the same time, seven plants were co-infected with GLRaV-3 and either GLRaV-1 or GLRaV-2. GVA was less common (24%).

A bioinformatics analysis of HTS data made it possible to assemble 123 complete or nearly complete viral genomes and 64 complete viroid genomes. Despite their wide distribution in samples, the smallest number of complete genomes was assembled for viruses of the genus *Marafivirus* (GRVFV, GAMaV, GSyV-1). For GFkV, in spite of the high coverage of individual fragments of the reference sequence, not a single complete genome was assembled. At the same time, the long genomes of GLRaVs (about 16,000–18,000 nt) were completely assembled.

Previously, viromes of ampelographic collections from two other regions of the Russian Federation, the Krasnodar Krai and the Republic of Dagestan, were examined [21,22]. A map of these regions is shown in Supplementary Figure S24. Based on the results of this study, it is worth noting that 12 species of viruses and viroids were found in all three collections (GVA, GLRaV-1, GRSPaV, GPGV, GRVFV, GFkV, GRGV, GVT, GV-Sat, HSVd, AGVd, and GYSVd-1). Among them, there are two economically significant pathogens: GVA and GLRaV-1. Compared to previous studies, grapevines from the Don ampelographic collection were found to be infected with GLRaV-3 and GVA to a greater extent.

## 4. Conclusions

The prevalent practice of using international grapevine varieties in present-day viticulture is being replaced by a trend of preserving the genetic diversity of local varieties, which possess unique characteristics and are able to reach their full potential within the specific environmental conditions of their native regions. Therefore, the metaviromic research on Russian germplasm is important for improving integrated control methods, limiting the spread of viral diseases, and improving the phytosanitary condition of promising grapevine varieties. The HTS data obtained in this study on the species and genetic composition of viruses can be applied in the development of highly sensitive and specific test systems for widespread use in commercial vineyards, germplasm collections, and for certifying planting materials.

Furthermore, information on the virome of grapevine germplasm is essential for improving biotechnological methods for in vitro propagation of virus-free grapevines. This is particularly important to protect unique local varieties and hybrids with high breeding potential.

This study expands international knowledge of the distribution and genetic diversity of grapevine viruses, especially considering the newly discovered species. Conducting further research is necessary to determine the biological and epidemiological characteristics of the novel viruses, including their distribution, transmission, interaction with the host plant, and their significance for the agroecosystem. 

## Figures and Tables

**Table 1:** Pairwise nucleotide sequence comparisons for three novel grapevine umbra-like viruses, %.

	GULV-2	GULV-3
GULV-3	44.5	100
GULV-4	48.4	44.3

**Table 2:** Pairwise nucleotide sequence comparisons for GULVs, umbraviruses, and ulaRNAs, %.

Group	Sequence	GULV-2	GULV-3	GULV-4
Umbraviruses	Genome	47.2–49.1	46.9–49.1	46.6–49.5
	RdRp	49.8–52.7	48.4–51.7	48.6–52.9
UlaRNAs	Genome	43.5–50.0	43.7–49.1	44.2–49.9
	RdRp	47.4–50.6	47.6–50.5	47.6–51.5

## Data Availability

Representative sequences were deposited in GenBank under the accession numbers OR834485-OR834512, OR872646-OR872652, OR892365-OR892525, OR947505-OR947518.

## Supplementary Materials

The following supporting information can be downloaded at: https://www.mdpi.com/article/10.3390/v15122429/s1, Figure S1: Phylogenetic tree based on genome sequences of grapevine leafroll-associated virus 1 (GLRaV-1) isolates obtained in this study, other Russian isolate and world isolates; Figure S2: Phylogenetic tree based on genome sequences of grapevine leafroll-associated virus 2 (GLRaV-2) isolates obtained in this study, other Russian isolates and world isolates; Figure S3: Phylogenetic tree based on genome sequences of grapevine leafroll-associated virus 3 (GLRaV-3) isolates obtained in this study, other Russian isolates and world isolates; Figure S4: Phylogenetic tree based on nucleotide sequences of RNA1 of grapevine fanleaf virus (GFLV) isolates obtained in this study, other Russian isolates and world isolates; Figure S5: Phylogenetic tree based on nucleotide sequences of RNA2 of grapevine fanleaf virus (GFLV) isolates obtained in this study, other Russian isolates and world isolates; Figure S6: Phylogenetic tree based on genome sequences of grapevine Pinot gris virus (GPGV) isolates obtained in this study, other Russian isolates and world isolates; Figure S7: Phylogenetic tree based on genome sequences of grapevine virus A (GVA) isolates obtained in this study, other Russian isolates and world isolates; Figure S8: Phylogenetic tree based on genome sequences of grapevine virus B (GVB) isolates obtained in this study, other Russian isolate and world isolates; Figure S9: Phylogenetic tree based on genome sequences of grapevine virus F (GVF) isolates obtained in this study, other Russian isolate and world isolates; Figure S10: Phylogenetic tree based on genome sequences of grapevine virus T (GVT) isolates obtained in this study, other Russian isolates and world isolates; Figure S11: Phylogenetic tree based on genome sequences of grapevine rupestris stem pitting-associated virus (GRSPaV) isolates obtained in this study, other Russian isolates and world isolates; Figure S12: Phylogenetic tree based on genome sequences of grapevine Syrah virus 1 (GSyV-1) isolates obtained in this study and world isolates; Figure S13: Phylogenetic tree based on genome sequences of grapevine rupestris vein feathering virus (GRVFV) isolates obtained in this study and world isolates; Figure S14: Phylogenetic tree based on genome sequences of grapevine-associated tymo-like virus (GaTLV) isolates obtained in this study and world isolates; Figure S15: Phylogenetic tree based on nucleotide sequences of RNA1 (a) and RNA2 (b) of Vitis cryptic virus (VCV) isolates obtained in this study, other Russian isolates and world isolates; Figure S16: Phylogenetic tree based on genome sequences of grapevine umbra-like virus (GULV) isolates obtained in this study and other Russian isolates; Figure S17: Phylogenetic tree based on genome sequences of hop stunt viroid (HSVd) isolates obtained in this study and world isolates; Figure S18: Phylogenetic tree based on genome sequences of grapevine yellow speckle viroid 1 (GYSVd-1) isolates obtained in this study, other Russian isolates and world isolates; Figure S19: Phylogenetic tree based on genome sequences of grapevine yellow speckle viroid 2 (GYSVd-2) isolates obtained in this study, other Russian isolates and world isolates; Figure S20: Phylogenetic tree based on genome sequences of Australian grapevine viroid (AGVd) isolates obtained in this study, other Russian isolates and world isolates; Figure S21: Phylogenetic tree based on nucleotide sequences of RNA1 of GAPV, alphapartitiviruses and unclassified partitiviruses; Figure S22: Phylogenetic tree based on amino acid sequences of the Pro-Pol region of GSV and members of the genera *Nepovirus, Fabavirus,* and *Comovirus*; Figure S23: Phylogenetic tree based on CP amino acid sequences of GSV and members of the genera *Nepovirus, Fabavirus,* and *Comovirus*; Figure S24. Map of the southern regions of Russia; Table S1: Basic information of selected grape samples and viral infection; Table S2: Complete sequences of viruses and viroids uploaded into GenBank with their identifier; Table S3: List of the RT-PCR primers used for virus and viroid detection; Table S4: List of sequences after Sanger sequencing submitted to the GenBank; Table S5: Initial data for phylogenetic analysis; Table S6: Number of reads and contigs obtained for each library after sequencing by Illumina NovaSeq 6000 Sequencing System; Table S7: Bioinformatics analysis and its validation; Table S8: Bioinformatics analysis: mapping of preprocessed reads to closest genome; Table S9: Viruses detected in GV-Sat positive samples; Table S10: The pairwise identity matrix of aa RdRp of GAPV and 21 closest partitiviruses; Table S11: The pairwise identity matrix of aa CP of GAPV and 18 closest partitiviruses; Table S12: The pairwise identity matrix of aa Pro-Pol region of GSV and 16 closest fabaviruses; Table S13: The pairwise identity matrix of aa CP of GSV and 16 closest fabaviruses; Table S14: The pairwise identity matrix of aa ORF3 and ORF4 of GULVs, ulaRNAs, and umbraviruses Table S15: The pairwise identity matrix of nt genome sequences of GULVs and 29 umbraviruses and ulaRNAs; Table S16: The pairwise identity matrix of nt RdRp of GULVs and 29 umbraviruses and ulaRNAs.
