# Supplementary material for: Grapevine Virome of the Don Ampelographic Collection in Russia Has Concealed Five Novel Viruses
Source: Viruses. 2023 Dec 14;15(12):2429. doi: 10.3390/v15122429 (PMC10747563; doi:10.3390/v15122429)
Supplement: Supplementary file 1 [file viruses-15-02429-s001.zip › Supplementary Figures.pdf]

# Grapevine Virome of the Don Ampelographic Collection in Russia Has Concealed Five Novel Viruses

**Daria Belkina[1,2], Daria Karpova[1,2], Elena Porotikova[1], Ilya Lifanov[1] and Svetlana Vinogradova[1,2]***

[1]Skryabin Institute of Bioengineering, Research Center of Biotechnology of the Russian Academy of Sciences, Leninsky Prospect, 33, Build. 2, 119071 Moscow, Russia
[2]North Caucasian Federal Scientific Center of Horticulture, Viticulture, Winemaking, 40 years of Victory street, Build. 39, 350901 Krasnodar, Russia

* Correspondence: coatprotein@bk.ru

## Supplementary Materials

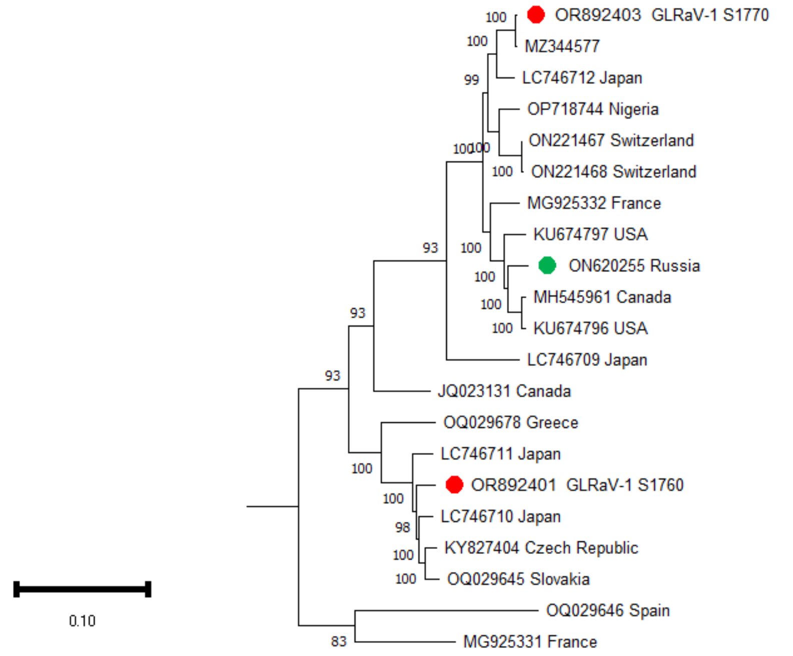


**Supplementary Figure S1.** Phylogenetic tree based on complete genome sequences of grapevine leafroll -associated virus 1 (GLRaV-1) isolates obtained in this study (red dots), other Russian isolate (green dot) and world isolates. Tree was constructed in MEGA11 using the maximum-likelihood method and GTR model with 1000 bootstrap replicates.

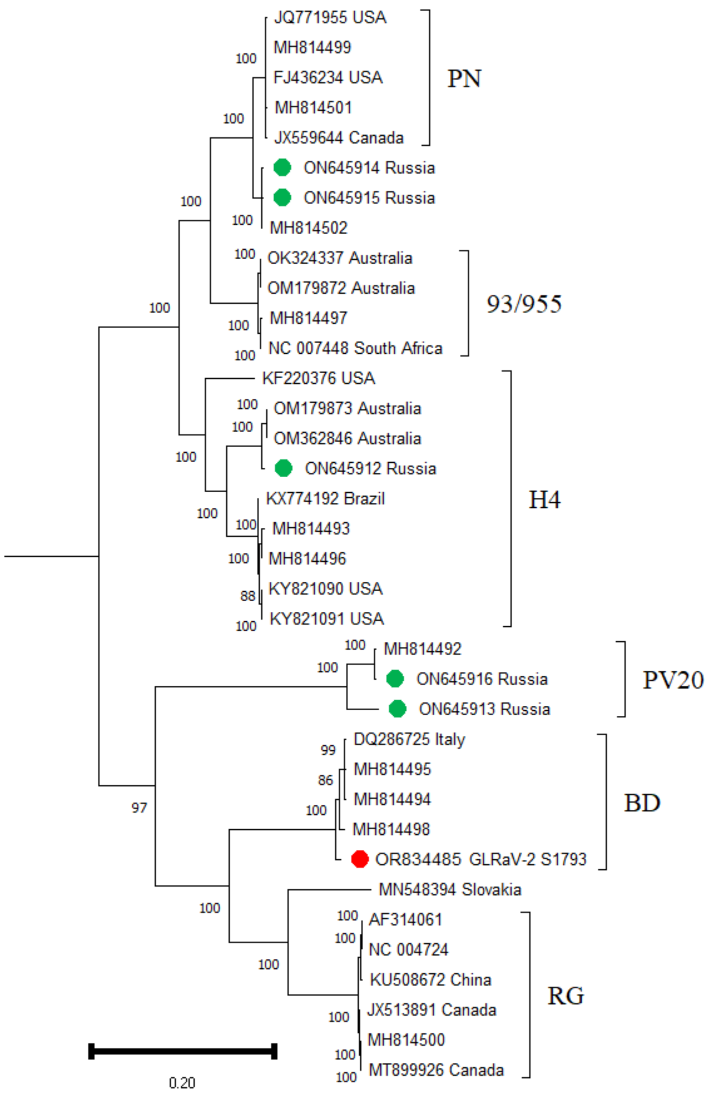


**Supplementary Figure S2.** Phylogenetic tree based on complete genome sequences of grapevine leafroll -associated virus 2 (GLRaV-2) isolates obtained in this study (red dot), other Russian isolates (green dots) and world isolates. Tree was constructed in MEGA11 using the maximum-likelihood method and GTR model with 1000 bootstrap replicates.

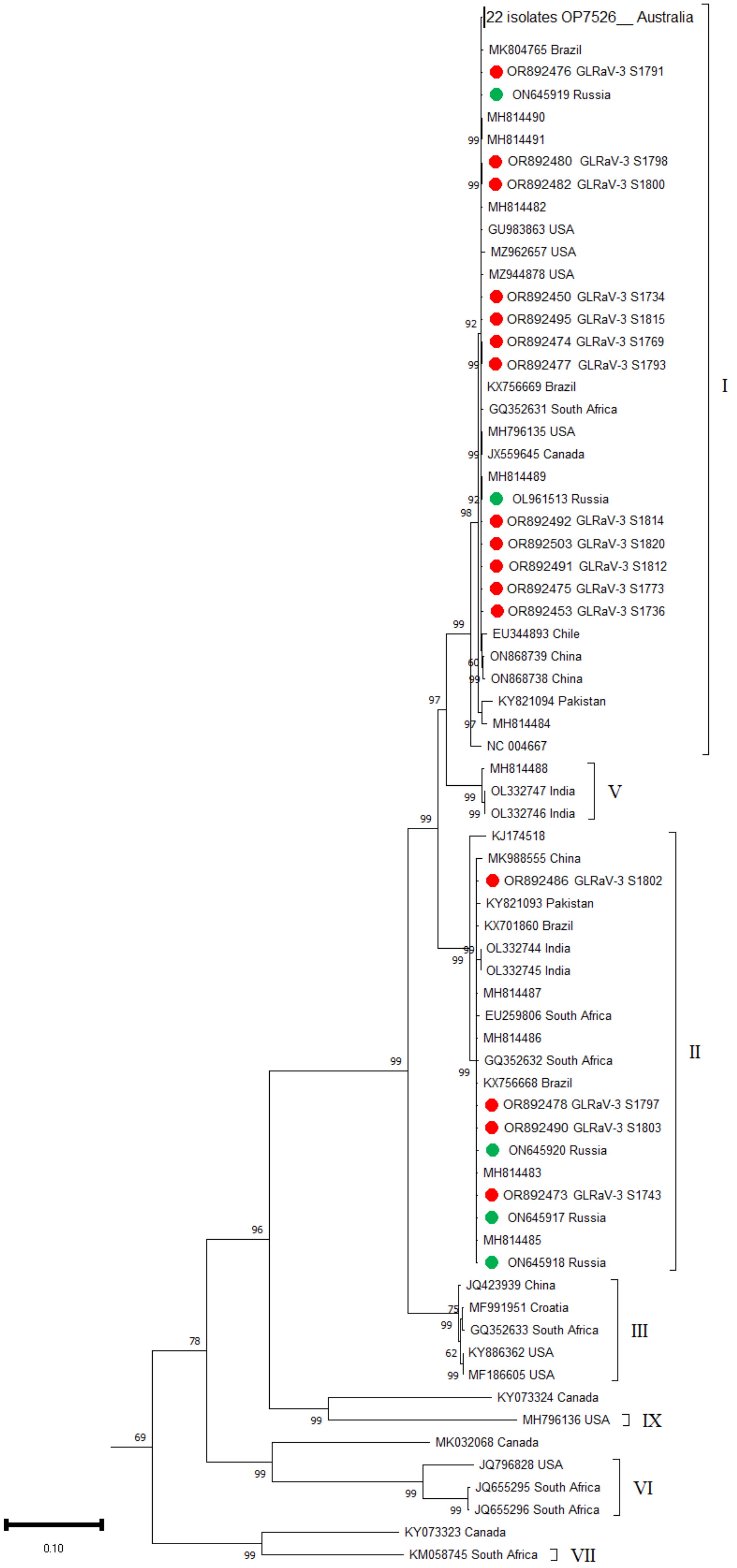


**Supplementary Figure S3.** Phylogenetic tree based on complete genome sequences of grapevine leafroll -associated virus 3 (GLRaV-3) isolates obtained in this study (red dots), other Russian isolates (green dots) and world isolates. Tree was constructed in MEGA11 using the maximum-likelihood method and GTR model with 1000 bootstrap replicates.

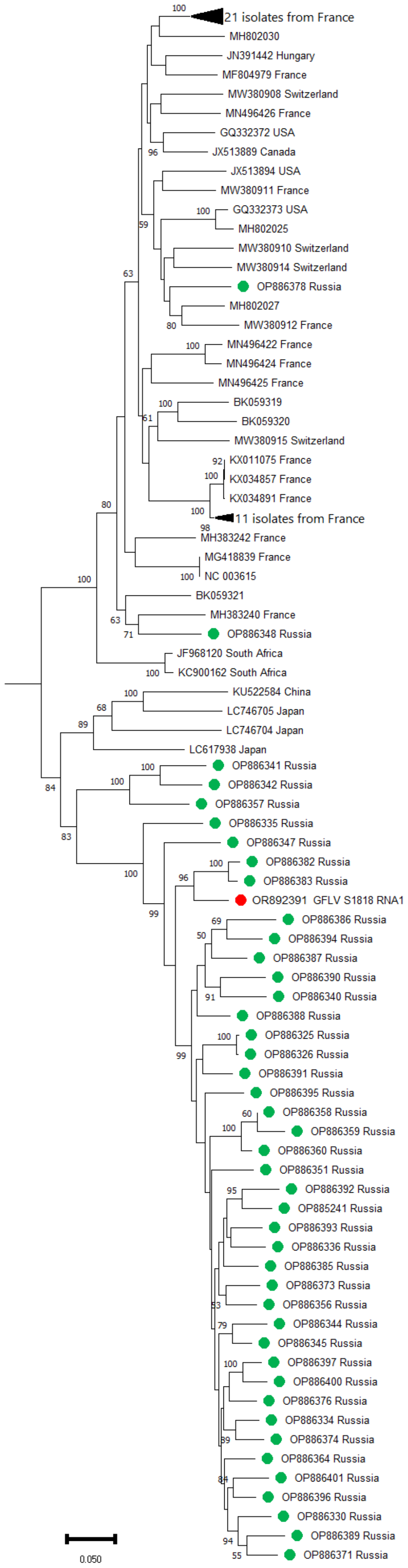


**Supplementary Figure S4.** Phylogenetic tree based on complete nucleotide sequences of RNA1 of grapevine fanleaf virus (GFLV) isolates obtained in this study (red dot), other Russian isolates (green dots) and world isolates. Tree was constructed in MEGA11 using the maximum-likelihood method and GTR model with 1000 bootstrap replicates. Bootstrap values below 50 are not shown.

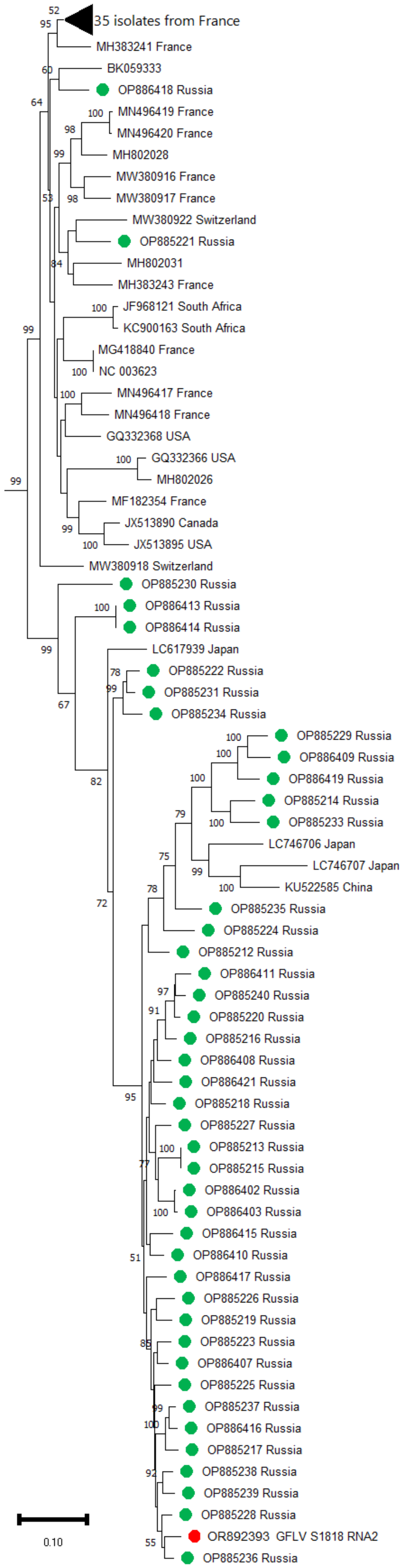


**Supplementary Figure S5.** Phylogenetic tree based on complete nucleotide sequences of RNA2 of grapevine fanleaf virus (GFLV) isolates obtained in this study (red dot), other Russian isolates (green dots) and world isolates. Tree was constructed in MEGA11 using the maximum-likelihood method and GTR model with 1000 bootstrap replicates. Bootstrap values below 50 are not shown.

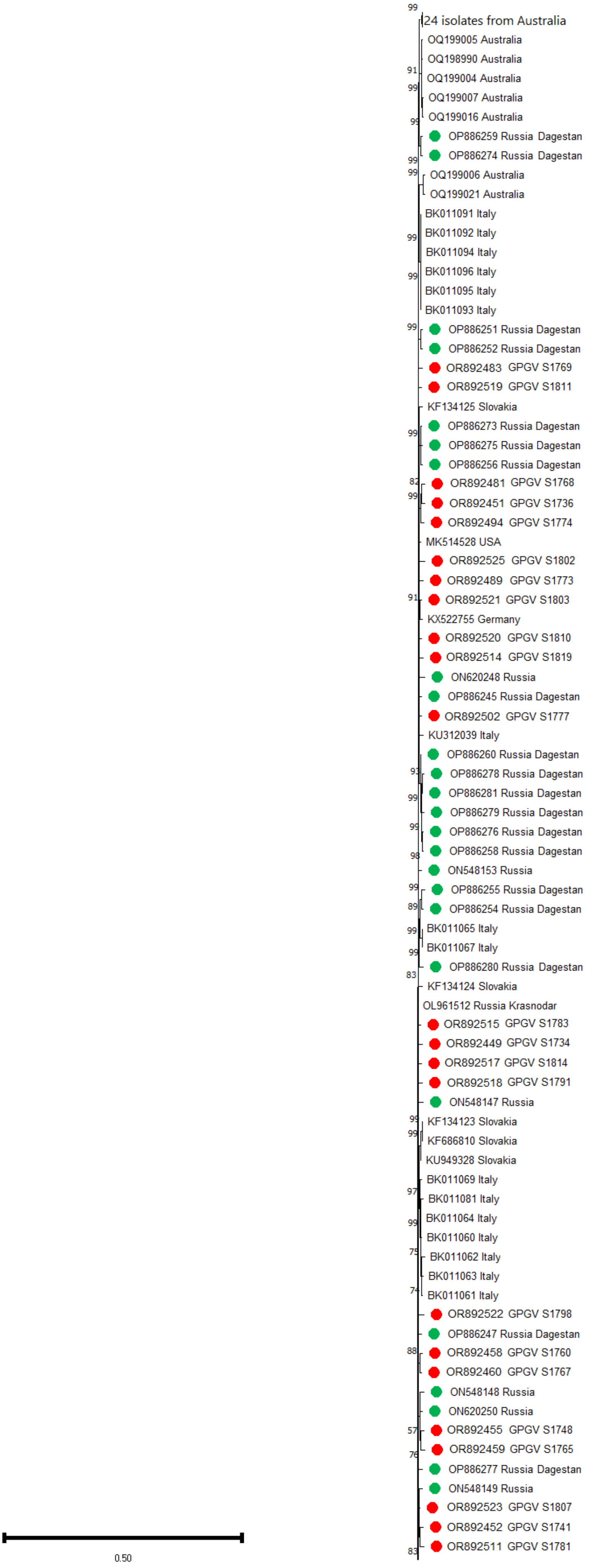
24 isolates from Australia
OQ199005 Australia
OQ198990 Australia
OQ199004 Australia
OQ199007 Australia
OQ199016 Australia
OP886259 Russia Dagestan
OP886274 Russia Dagestan
OQ199006 Australia
OQ199021 Australia
BK011091 Italy
BK011092 Italy
BK011094 Italy
BK011096 Italy
BK011095 Italy
BK011093 Italy
OP886251 Russia Dagestan
OP886252 Russia Dagestan
OR892483 GPGV S1769
OR892519 GPGV S1811
KF134125 Slovakia
OP886273 Russia Dagestan
OP886275 Russia Dagestan
OP886256 Russia Dagestan
OR892481 GPGV S1768
OR892451 GPGV S1736
OR892494 GPGV S1774
MK514528 USA
OR892525 GPGV S1802
OR892489 GPGV S1773
OR892521 GPGV S1803
KX522755 Germany
OR892520 GPGV S1810
OR892514 GPGV S1819
ON620248 Russia
OP886245 Russia Dagestan
OR892502 GPGV S1777
KU312039 Italy
OP886260 Russia Dagestan
OP886278 Russia Dagestan
OP886281 Russia Dagestan
OP886279 Russia Dagestan
OP886276 Russia Dagestan
OP886258 Russia Dagestan
ON548153 Russia
OP886255 Russia Dagestan
OP886254 Russia Dagestan
BK011065 Italy
BK011067 Italy
OP886280 Russia Dagestan
KF134124 Slovakia
OL961512 Russia Krasnodar
OR892515 GPGV S1783
OR892449 GPGV S1734
OR892517 GPGV S1814
OR892518 GPGV S1791
ON548147 Russia
KF134123 Slovakia
KF686810 Slovakia
KU949328 Slovakia
BK011069 Italy
BK011081 Italy
BK011064 Italy
BK011060 Italy
BK011062 Italy
BK011063 Italy
BK011061 Italy
OR892522 GPGV S1798
OP886247 Russia Dagestan
OR892458 GPGV S1760
OR892460 GPGV S1767
ON548148 Russia
ON620250 Russia
OR892455 GPGV S1748
OR892459 GPGV S1765
OP886277 Russia Dagestan
ON548149 Russia
OR892523 GPGV S1807
OR892452 GPGV S1741
OR892511 GPGV S1781
0.50

(a)

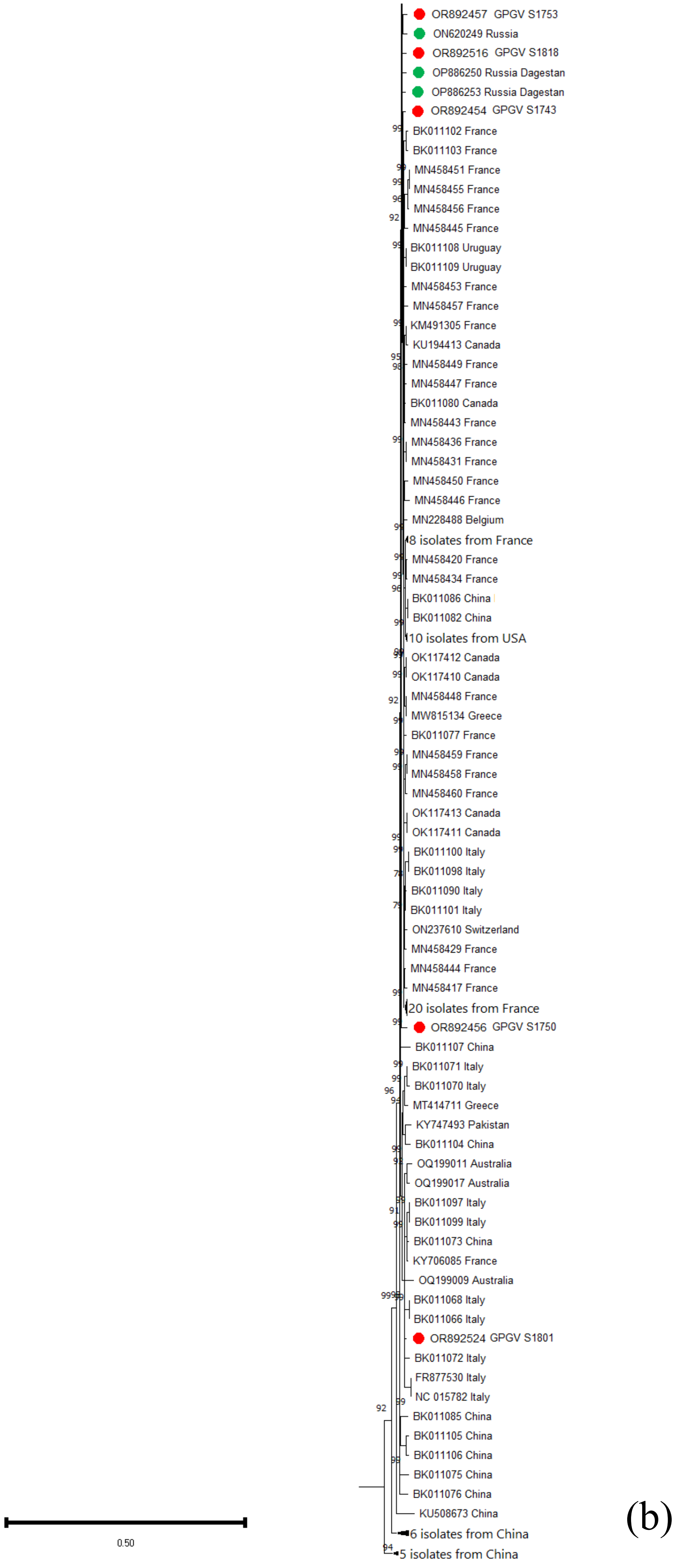


**Supplementary Figure S6 (a-b).** Phylogenetic tree based on complete genome sequences of grapevine Pinot gris virus (GPGV) isolates obtained in this study (red dots), other Russian isolates (green dots) and world isolates. Tree was constructed in MEGA11 using the maximum-likelihood method and GTR model with 1000 bootstrap replicates. Bootstrap values below 50 are not shown.

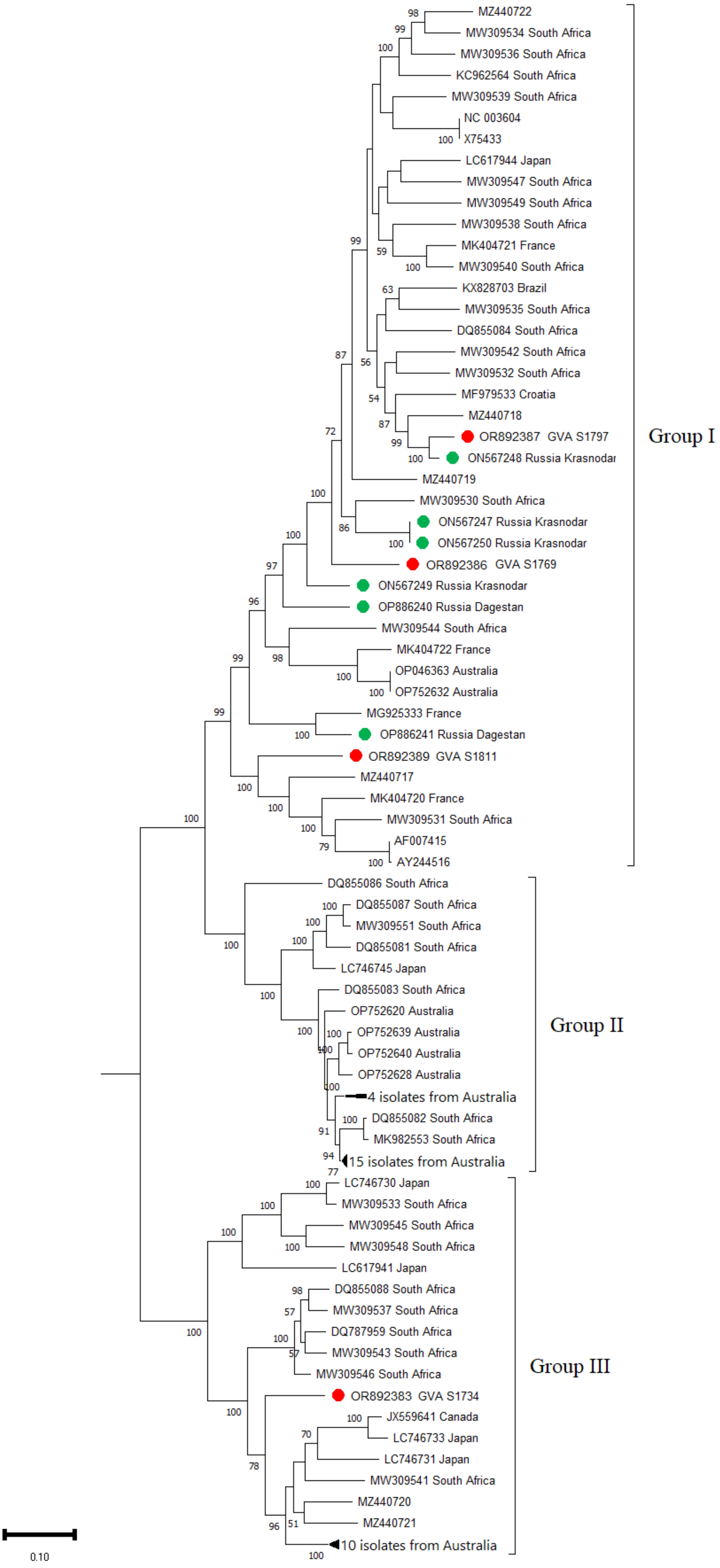


**Supplementary Figure S7.** Phylogenetic tree based on complete genome sequences of grapevine virus A (GVA) isolates obtained in this study (red dots), other Russian isolates (green dots) and world isolates. Tree was constructed in MEGA11 using the maximum-likelihood method and GTR model with 1000 bootstrap replicates. Bootstrap values below 50 are not shown.

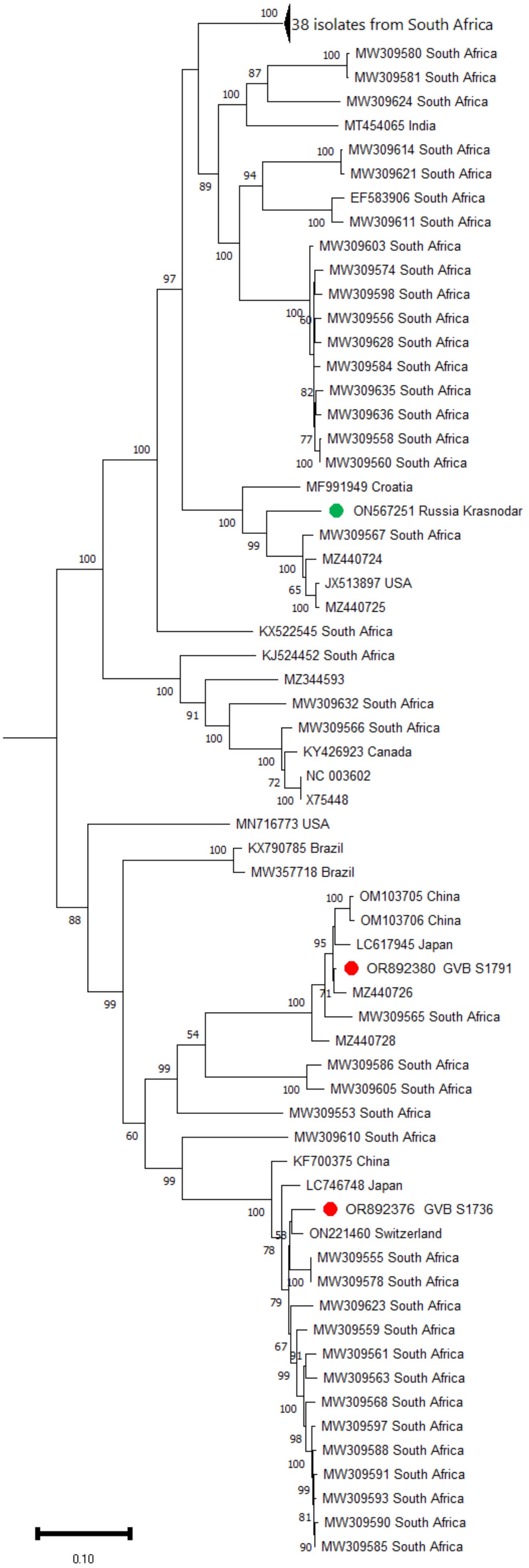


**Supplementary Figure S8.** Phylogenetic tree based on complete genome sequences of grapevine virus B (GVB) isolates obtained in this study (red dots), other Russian isolate (green dot) and world isolates. Tree was constructed in MEGA11 using the maximum-likelihood method and GTR model with 1000 bootstrap replicates. Bootstrap values below 50 are not shown.

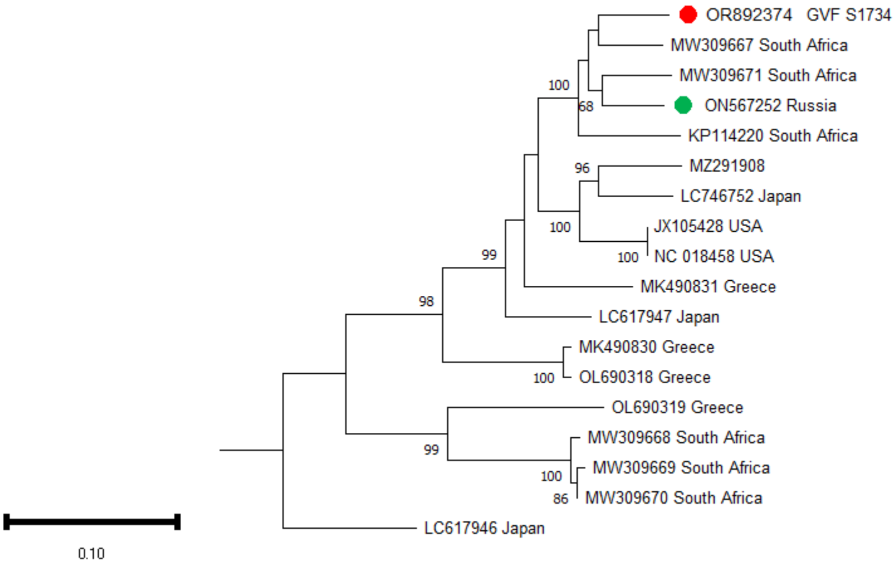


**Supplementary Figure S9.** Phylogenetic tree based on complete genome sequences of grapevine virus F (GVF) isolates obtained in this study (red dot), other Russian isolate (green dot) and world isolates. Tree was constructed in MEGA11 using the maximum-likelihood method and GTR model with 1000 boot-strap replicates. Bootstrap values below 50 are not shown.

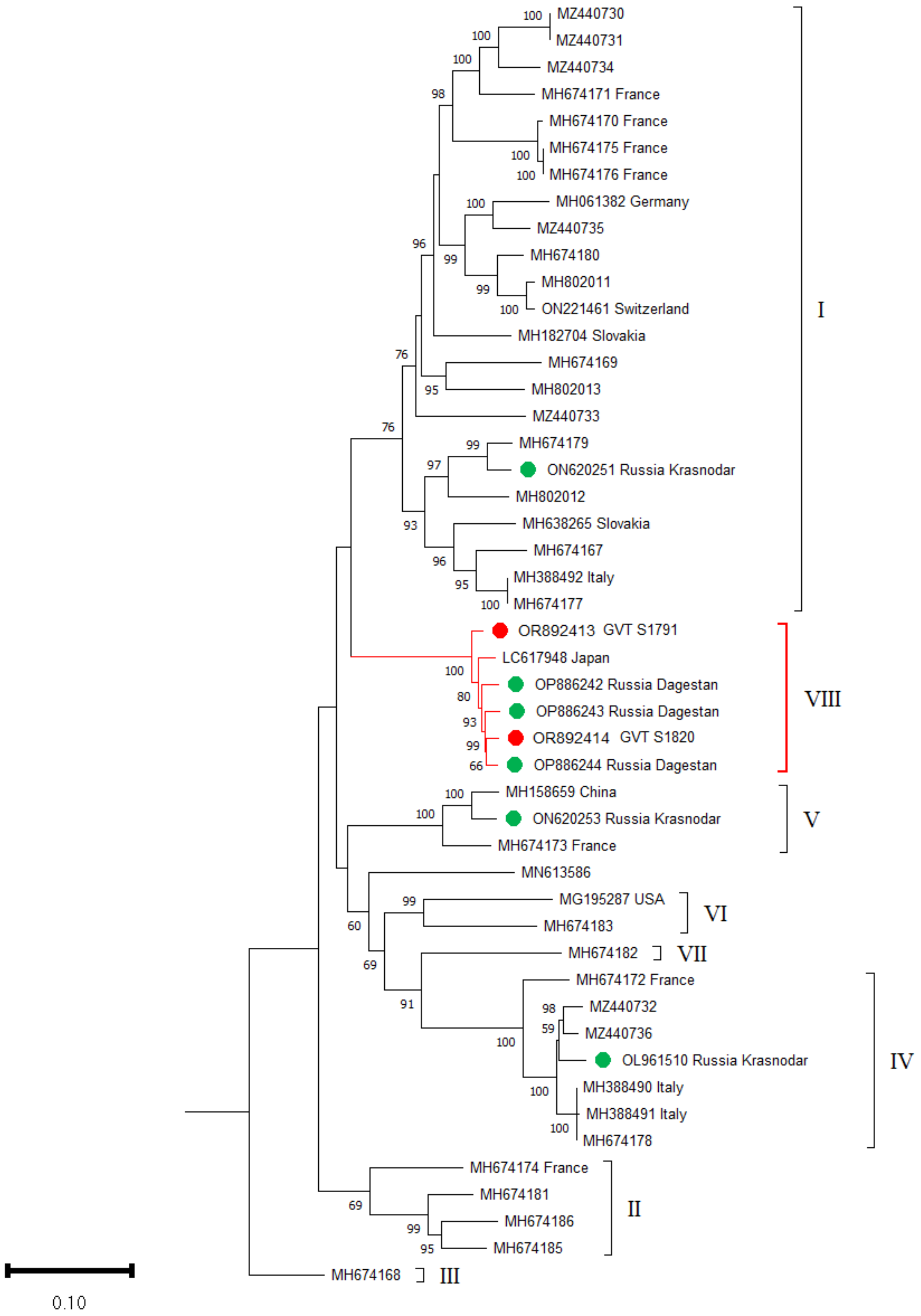


**Supplementary Figure S10.** Phylogenetic tree based on complete genome sequences of grapevine virus T (GVT) isolates obtained in this study (red dots), other Russian isolates (green dots) and world isolates. Tree was constructed in MEGA11 using the maximum-likelihood method and GTR model with 1000 bootstrap replicates. Bootstrap values below 50 are not shown.

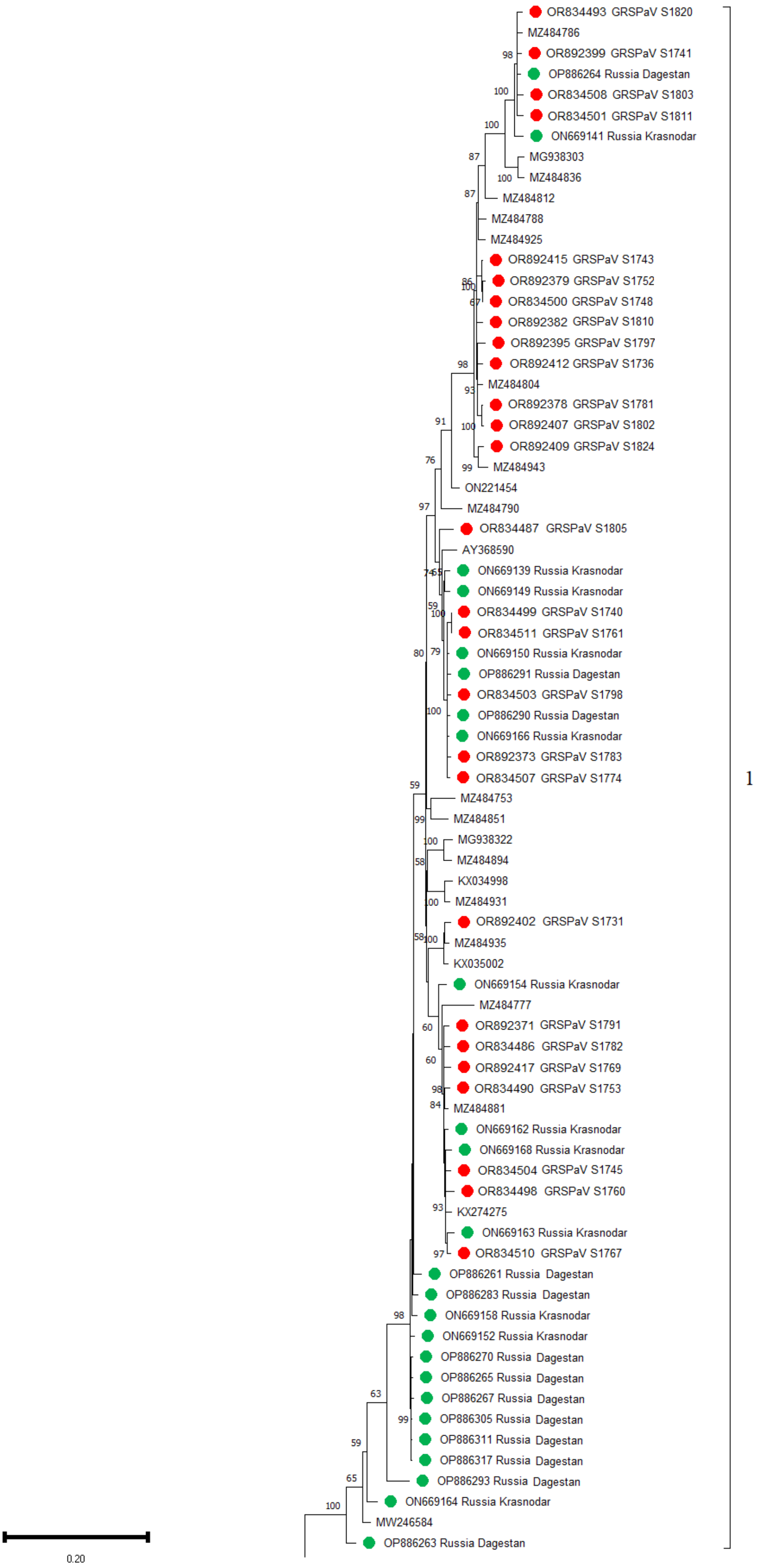

OR834493 GRSPaV S1820
MZ484786
OR892399 GRSPaV S1741
OP886264 Russia Dagestan
OR834508 GRSPaV S1803
OR834501 GRSPaV S1811
ON669141 Russia Krasnodar
MG938303
MZ484836
MZ484812
MZ484788
MZ484925
OR892415 GRSPaV S1743
OR892379 GRSPaV S1752
OR834500 GRSPaV S1748
OR892382 GRSPaV S1810
OR892395 GRSPaV S1797
OR892412 GRSPaV S1736
MZ484804
OR892378 GRSPaV S1781
OR892407 GRSPaV S1802
OR892409 GRSPaV S1824
MZ484943
ON221454
MZ484790
OR834487 GRSPaV S1805
AY368590
ON669139 Russia Krasnodar
ON669149 Russia Krasnodar
OR834499 GRSPaV S1740
OR834511 GRSPaV S1761
ON669150 Russia Krasnodar
OP886291 Russia Dagestan
OR834503 GRSPaV S1798
OP886290 Russia Dagestan
ON669166 Russia Krasnodar
OR892373 GRSPaV S1783
OR834507 GRSPaV S1774
MZ484753
MZ484851
MG938322
MZ484894
KX034998
MZ484931
OR892402 GRSPaV S1731
MZ484935
KX035002
ON669154 Russia Krasnodar
MZ484777
OR892371 GRSPaV S1791
OR834486 GRSPaV S1782
OR892417 GRSPaV S1769
OR834490 GRSPaV S1753
MZ484881
ON669162 Russia Krasnodar
ON669168 Russia Krasnodar
OR834504 GRSPaV S1745
OR834498 GRSPaV S1760
KX274275
ON669163 Russia Krasnodar
OR834510 GRSPaV S1767
OP886261 Russia Dagestan
OP886283 Russia Dagestan
ON669158 Russia Krasnodar
ON669152 Russia Krasnodar
OP886270 Russia Dagestan
OP886265 Russia Dagestan
OP886267 Russia Dagestan
OP886305 Russia Dagestan
OP886311 Russia Dagestan
OP886317 Russia Dagestan
OP886293 Russia Dagestan
ON669164 Russia Krasnodar
MW246584
OP886263 Russia Dagestan
1
0.20


(a)

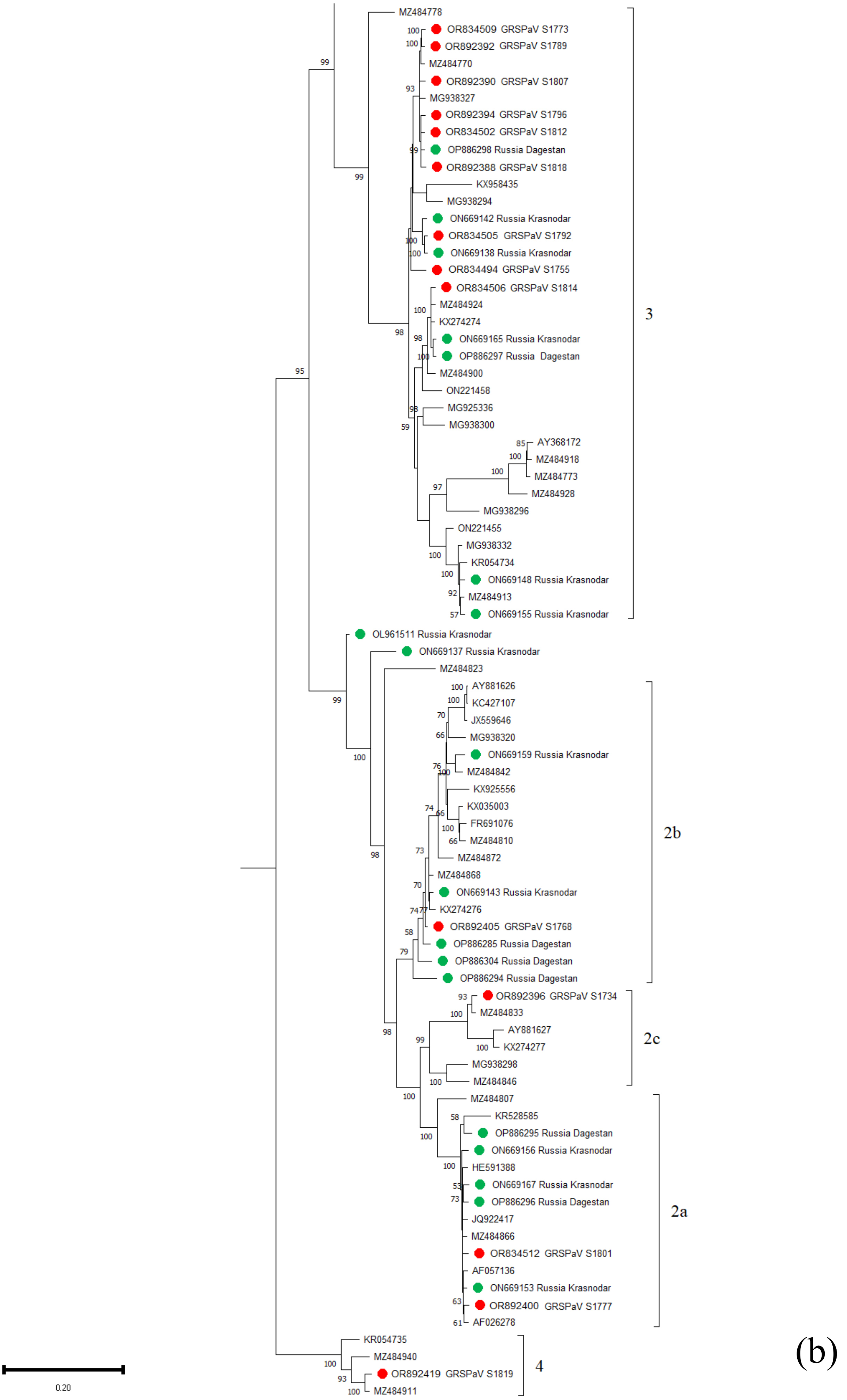


**Supplementary Figure S11 (a-b).** Phylogenetic tree based on complete genome sequences of grapevine rupestris stem pitting-associated virus (GRSPaV) isolates obtained in this study (red dots), other Russian isolates (green dots) and world isolates. Tree was constructed in MEGA11 using the maximum-likelihood method and GTR model with 1000 bootstrap replicates. Bootstrap values below 50 are not shown.

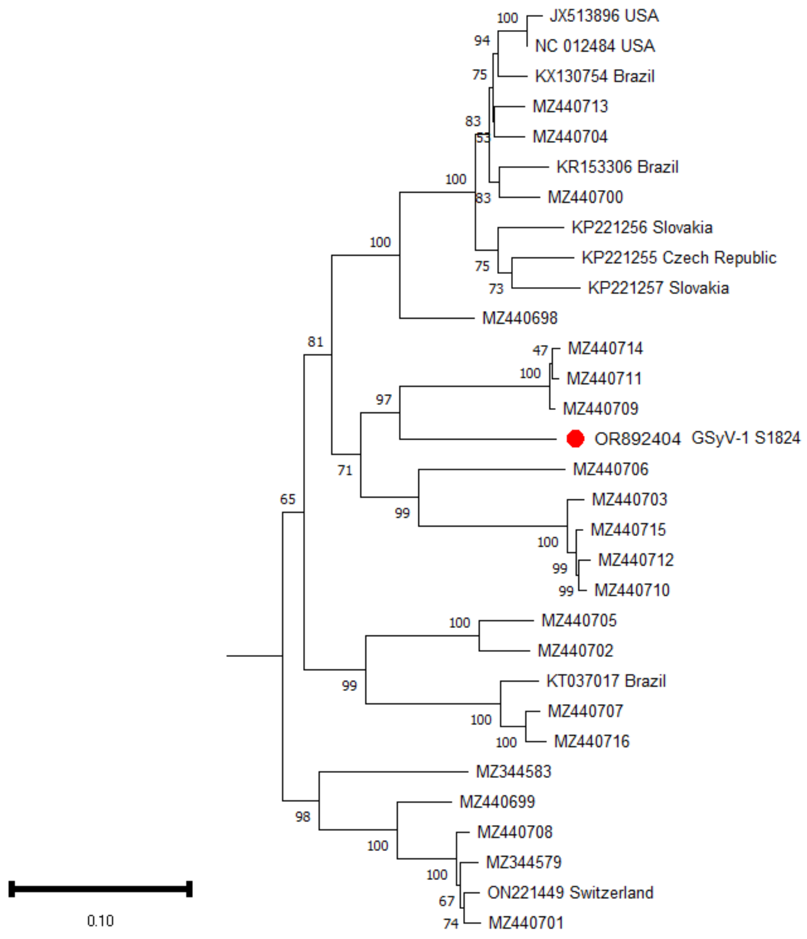


**Supplementary Figure S12.** Phylogenetic tree based on complete and nearly complete genome sequenc-es of grapevine Syrah virus 1 (GSyV-1) isolates obtained in this study (red dot) and world isolates. Tree was constructed in MEGA11 using the maximum-likelihood method and GTR model with 1000 boot-strap replicates. Bootstrap values below 50 are not shown.

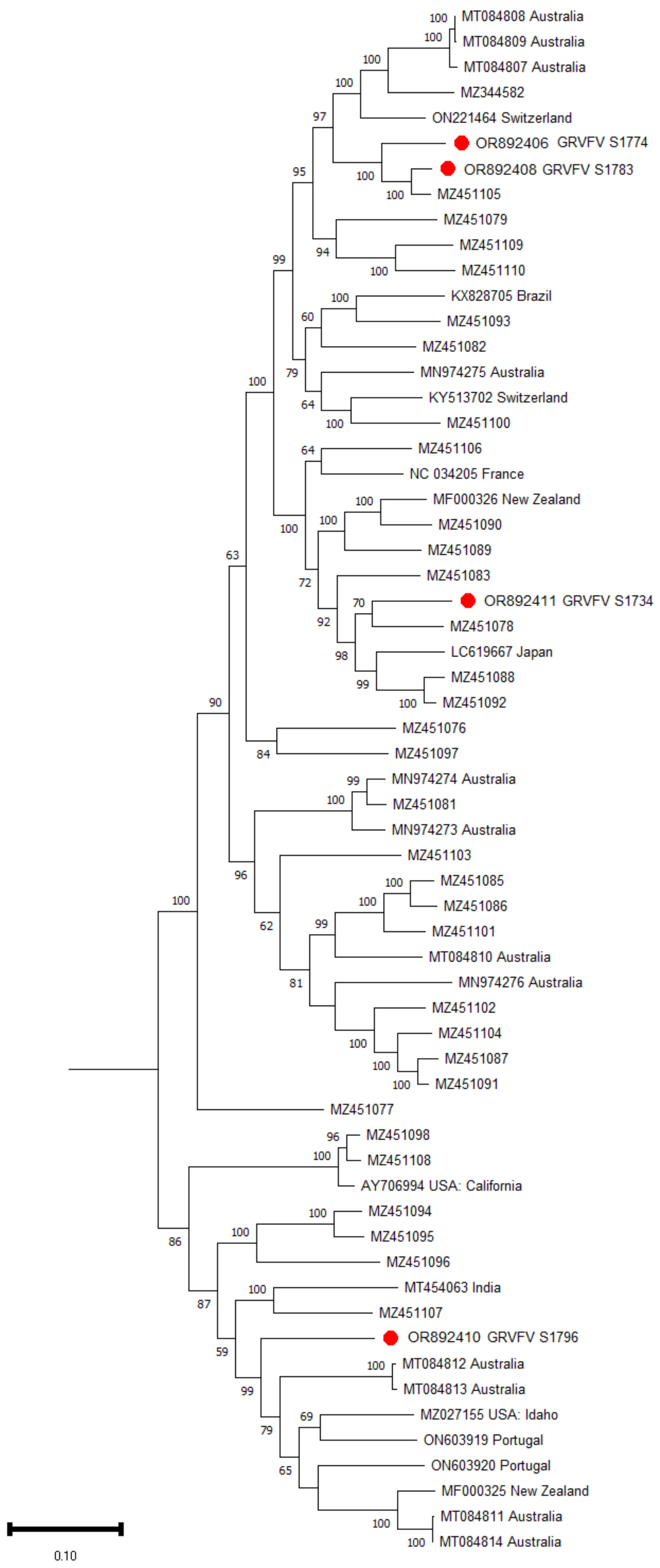


**Supplementary Figure S13.** Phylogenetic tree based on complete genome sequences of grapevine rupestris vein feathering virus (GRVFV) isolates obtained in this study (red dots) and world isolates. Tree was constructed in MEGA11 using the maximum-likelihood method and GTR model with 1000 bootstrap replicates. Bootstrap values below 50 are not shown.

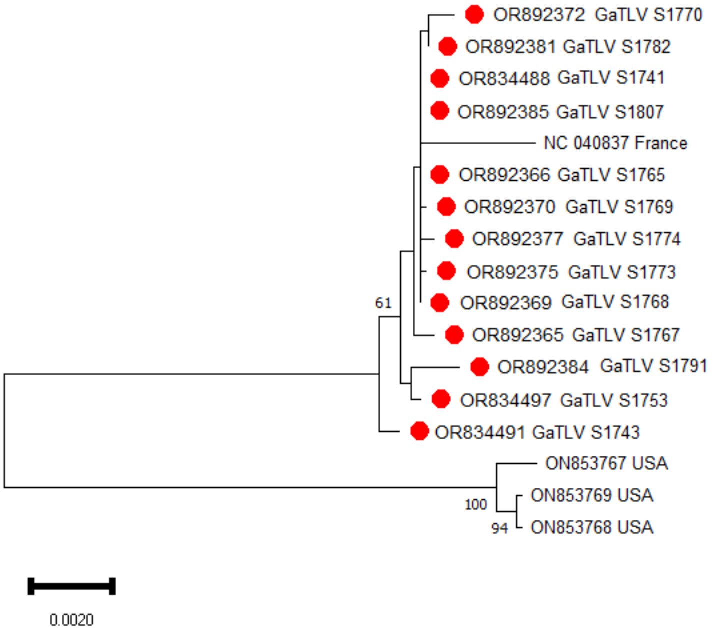


**Supplementary Figure S14.** Phylogenetic tree based on complete genome sequences of grapevine-associated tymo-like virus (GaTLV) isolates obtained in this study (red dots) and world isolates. Tree was constructed in MEGA11 using the maximum-likelihood method and GTR model with 1000 boot-strap replicates. Bootstrap values below 50 are not shown.

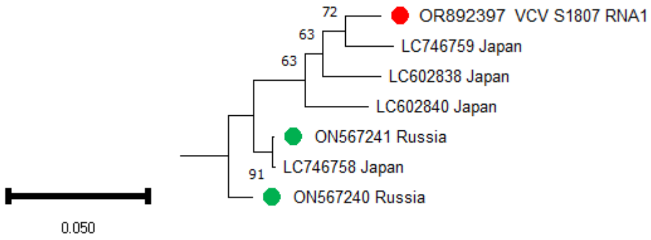


(a)

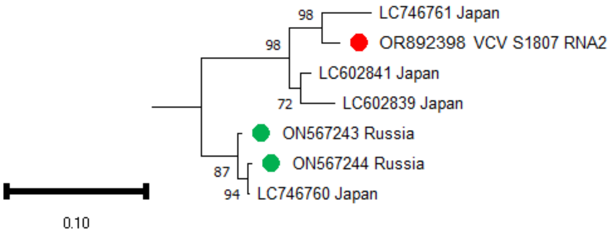


(b)

**Supplementary Figure S15 (a-b).** Phylogenetic tree based on complete nucleotide sequences of RNA1 (a) and RNA2 (b) of Vitis cryptic virus (VCV) isolates obtained in this study (red dot), other Russian isolates (green dots) and world isolates. Tree was constructed in MEGA11 using the maximum-likelihood method and GTR model with 1000 bootstrap replicates. Bootstrap values below 50 are not shown.

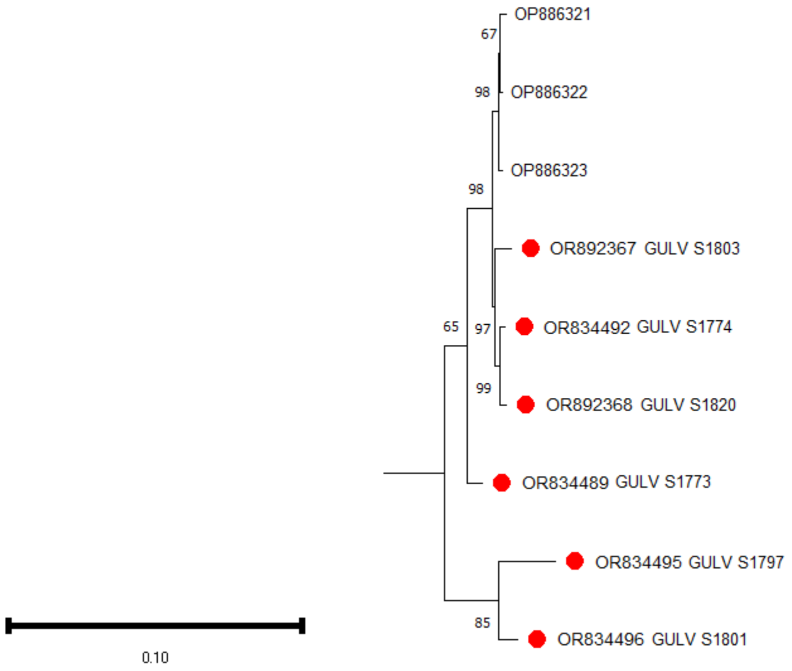


**Supplementary Figure S16.** Phylogenetic tree based on complete genome sequences of grapevine umbra -like virus (GULV) isolates obtained in this study (red dots) and other Russian isolates. Tree was con-structed in MEGA11 using the maximum-likelihood method and Tamura-Nei model with 1000 boot-strap replicates. Bootstrap values below 50 are not shown.

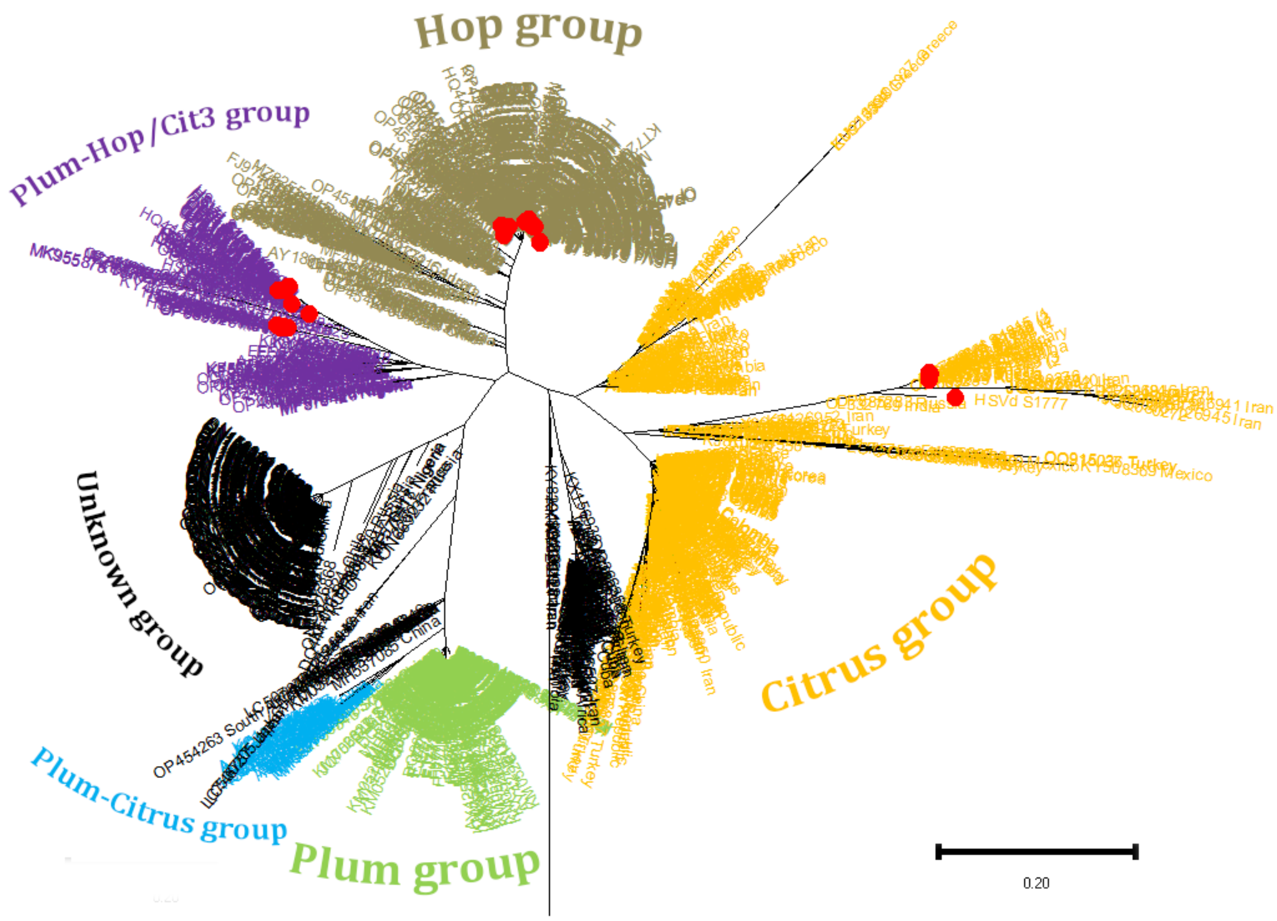


**Supplementary Figure S17.** Phylogenetic tree based on complete genome sequences of hop stunt viroid (HSVd) isolates obtained in this study (red dots) and world isolates. Tree was constructed in MEGA11 using the maximum-likelihood method and Tamura 3-parameter model with 1000 bootstrap replicates.

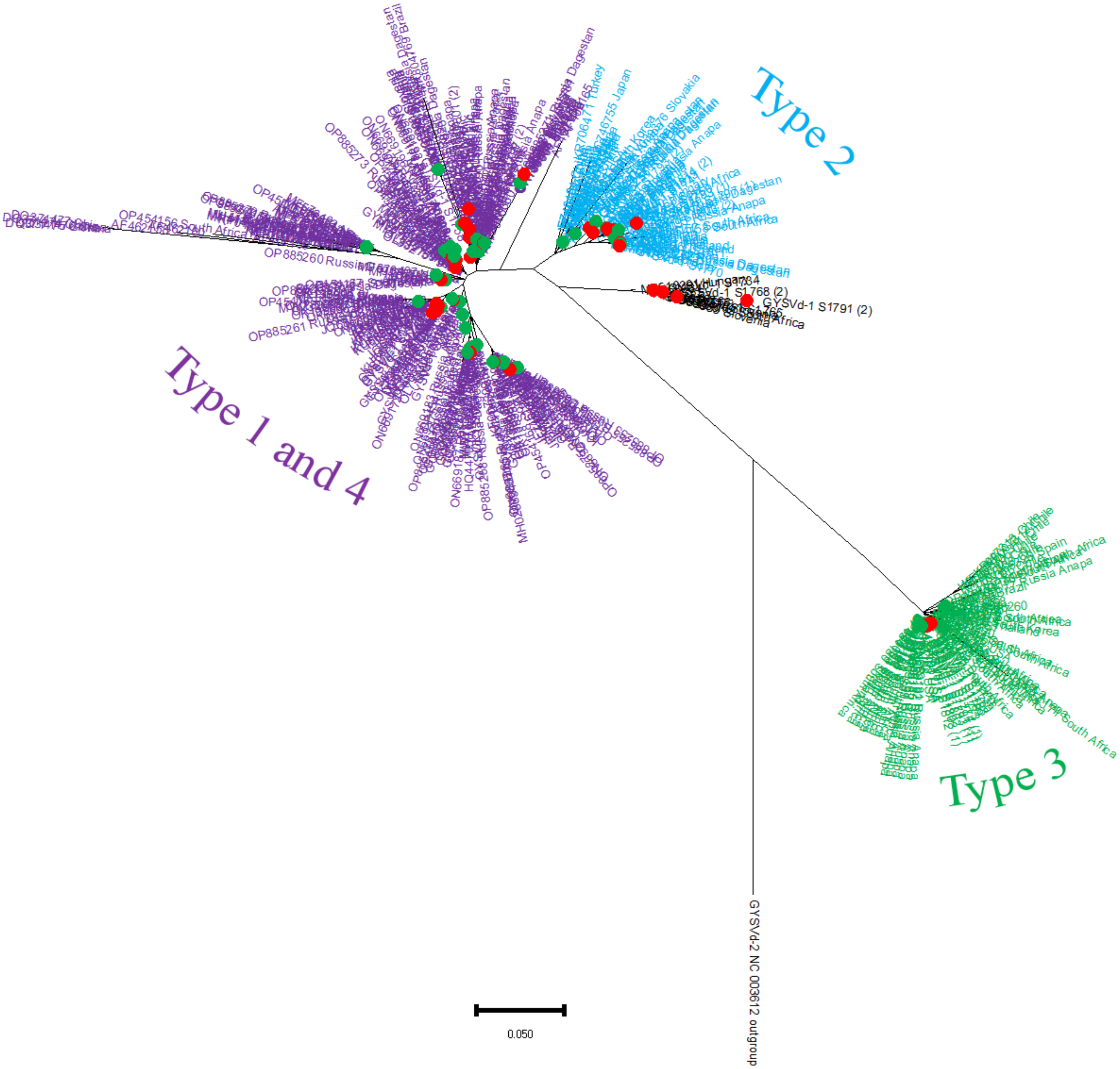


**Supplementary Figure S18.** Phylogenetic tree based on complete genome sequences of grapevine yel-low speckle viroid 1 (GYSVd-1) isolates obtained in this study (red dots), other Russian isolates (green dots) and world isolates. Tree was constructed in MEGA11 using the maximum-likelihood method and GTR model with 1000 bootstrap replicates.

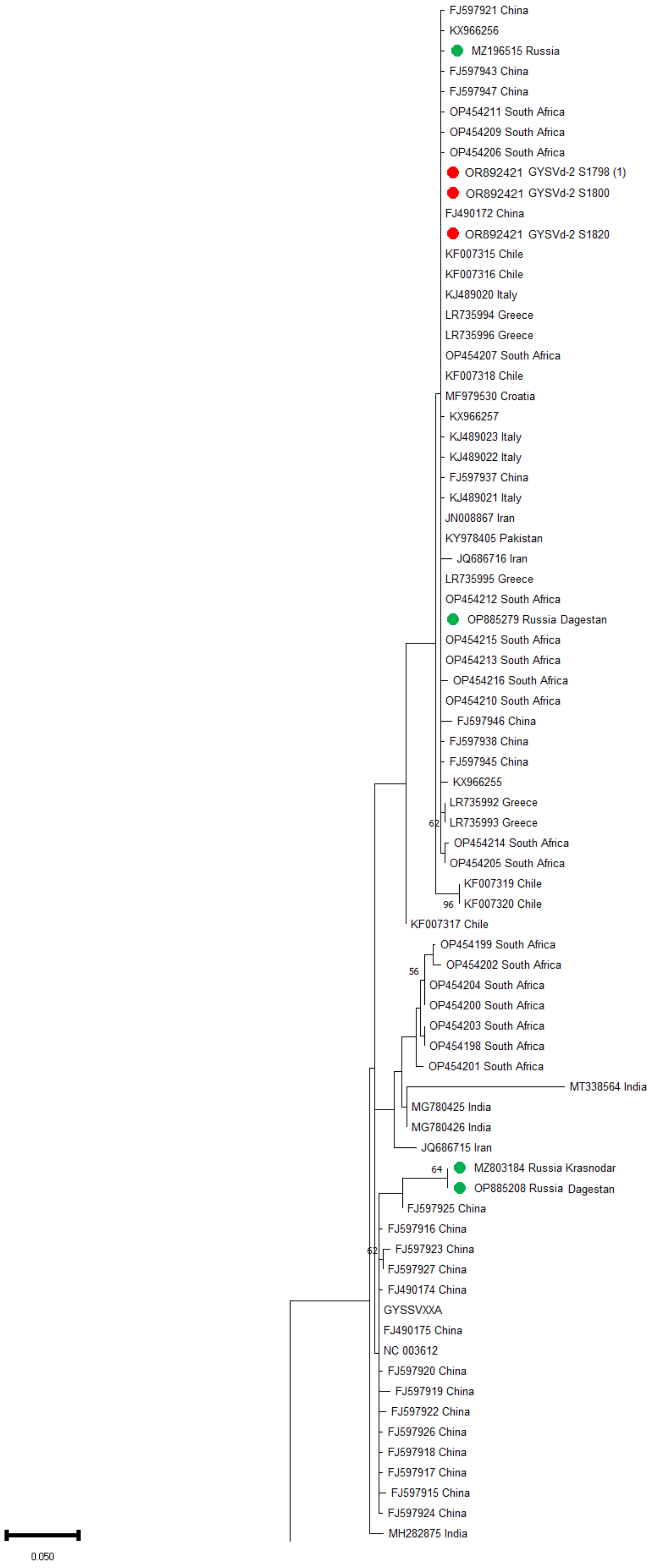
FJ597921 China
KX966256
MZ196515 Russia
FJ597943 China
FJ597947 China
OP454211 South Africa
OP454209 South Africa
OP454206 South Africa
OR892421 GYSVd-2 S1798 (1)
OR892421 GYSVd-2 S1800
FJ490172 China
OR892421 GYSVd-2 S1820
KF007315 Chile
KF007316 Chile
KJ489020 Italy
LR735994 Greece
LR735996 Greece
OP454207 South Africa
KF007318 Chile
MF979530 Croatia
KX966257
KJ489023 Italy
KJ489022 Italy
FJ597937 China
KJ489021 Italy
JN008867 Iran
KY978405 Pakistan
JQ686716 Iran
LR735995 Greece
OP454212 South Africa
OP885279 Russia Dagestan
OP454215 South Africa
OP454213 South Africa
OP454216 South Africa
OP454210 South Africa
FJ597946 China
FJ597938 China
FJ597945 China
KX966255
LR735992 Greece
62
LR735993 Greece
OP454214 South Africa
OP454205 South Africa
KF007319 Chile
96
KF007320 Chile
KF007317 Chile
OP454199 South Africa
56
OP454202 South Africa
OP454204 South Africa
OP454200 South Africa
OP454203 South Africa
OP454198 South Africa
OP454201 South Africa
MT338564 India
MG780425 India
MG780426 India
JQ686715 Iran
64
MZ803184 Russia Krasnodar
OP885208 Russia Dagestan
FJ597925 China
FJ597916 China
62
FJ597923 China
FJ597927 China
FJ490174 China
GYSSVXXA
FJ490175 China
NC 003612
FJ597920 China
FJ597919 China
FJ597922 China
FJ597926 China
FJ597918 China
FJ597917 China
FJ597915 China
FJ597924 China
MH282875 India
0.050
(a)

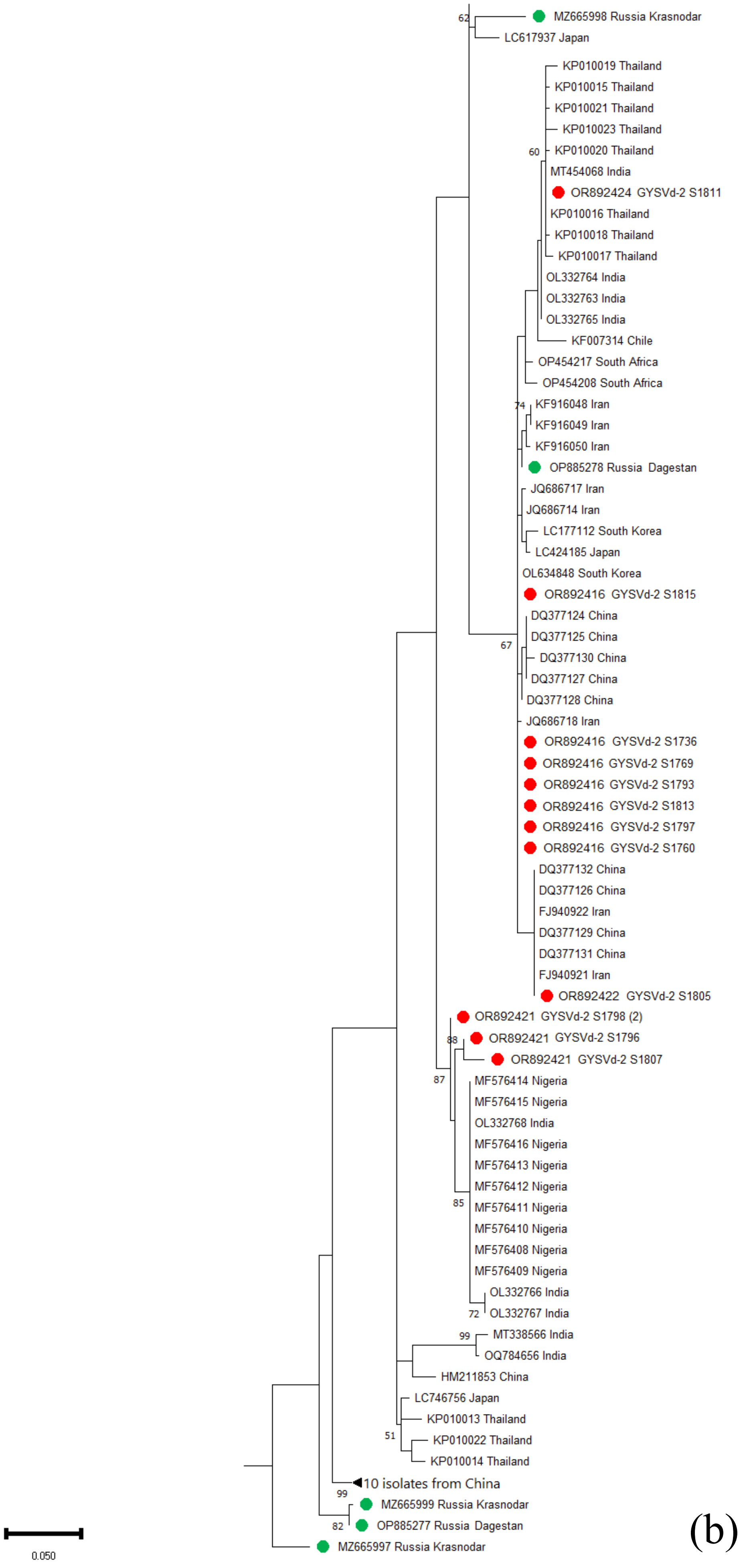


**Supplementary Figure S19 (a-b).** Phylogenetic tree based on complete genome sequences of grapevine yellow speckle viroid 2 (GYSVd-2) isolates obtained in this study (red dots), other Russian isolates (green dots) and world isolates. Tree was constructed in MEGA11 using the maximum-likelihood meth-od and GTR model with 1000 bootstrap replicates. Bootstrap values below 50 are not shown.

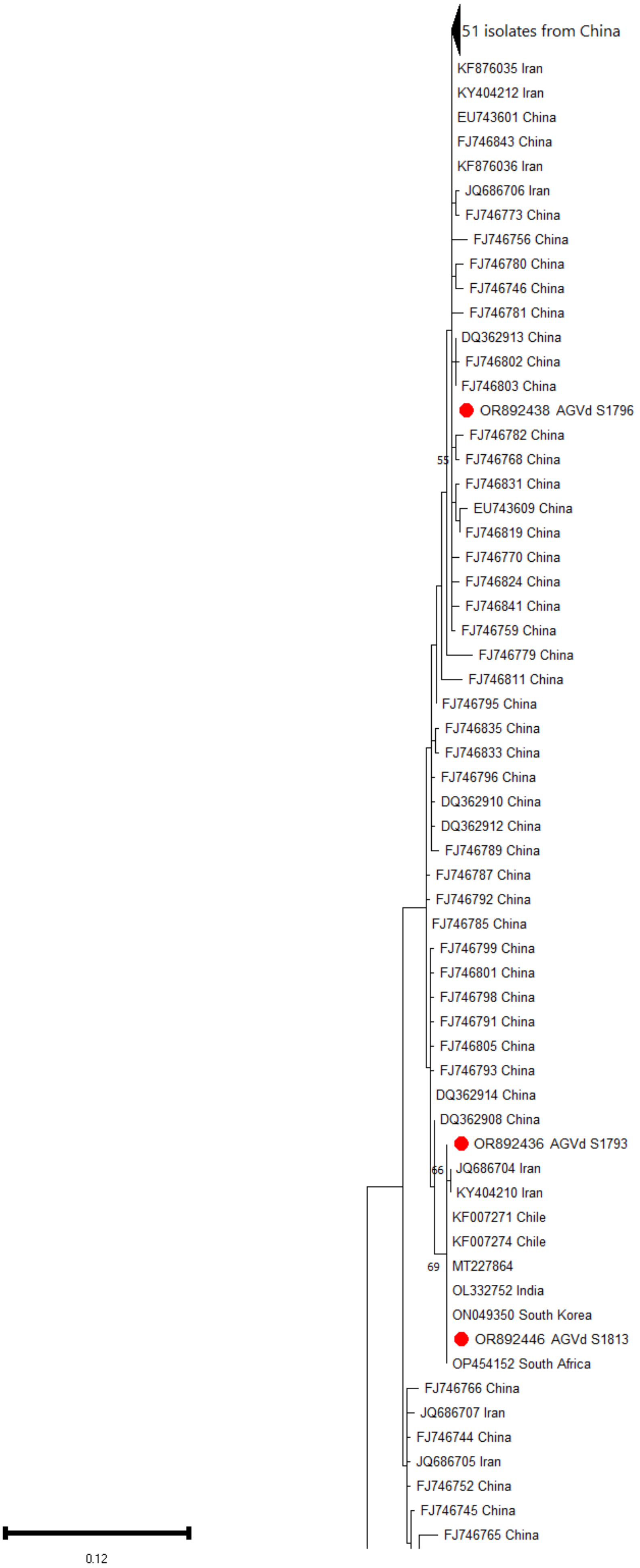
51 isolates from China
KF876035 Iran
KY404212 Iran
EU743601 China
FJ746843 China
KF876036 Iran
JQ686706 Iran
FJ746773 China
FJ746756 China
FJ746780 China
FJ746746 China
FJ746781 China
DQ362913 China
FJ746802 China
FJ746803 China
OR892438 AGVd S1796
FJ746782 China
55
FJ746768 China
FJ746831 China
EU743609 China
FJ746819 China
FJ746770 China
FJ746824 China
FJ746841 China
FJ746759 China
FJ746779 China
FJ746811 China
FJ746795 China
FJ746835 China
FJ746833 China
FJ746796 China
DQ362910 China
DQ362912 China
FJ746789 China
FJ746787 China
FJ746792 China
FJ746785 China
FJ746799 China
FJ746801 China
FJ746798 China
FJ746791 China
FJ746805 China
FJ746793 China
DQ362914 China
DQ362908 China
OR892436 AGVd S1793
66
JQ686704 Iran
KY404210 Iran
KF007271 Chile
KF007274 Chile
69
MT227864
OL332752 India
ON049350 South Korea
OR892446 AGVd S1813
OP454152 South Africa
FJ746766 China
JQ686707 Iran
FJ746744 China
JQ686705 Iran
FJ746752 China
FJ746745 China
FJ746765 China
0.12

(a)

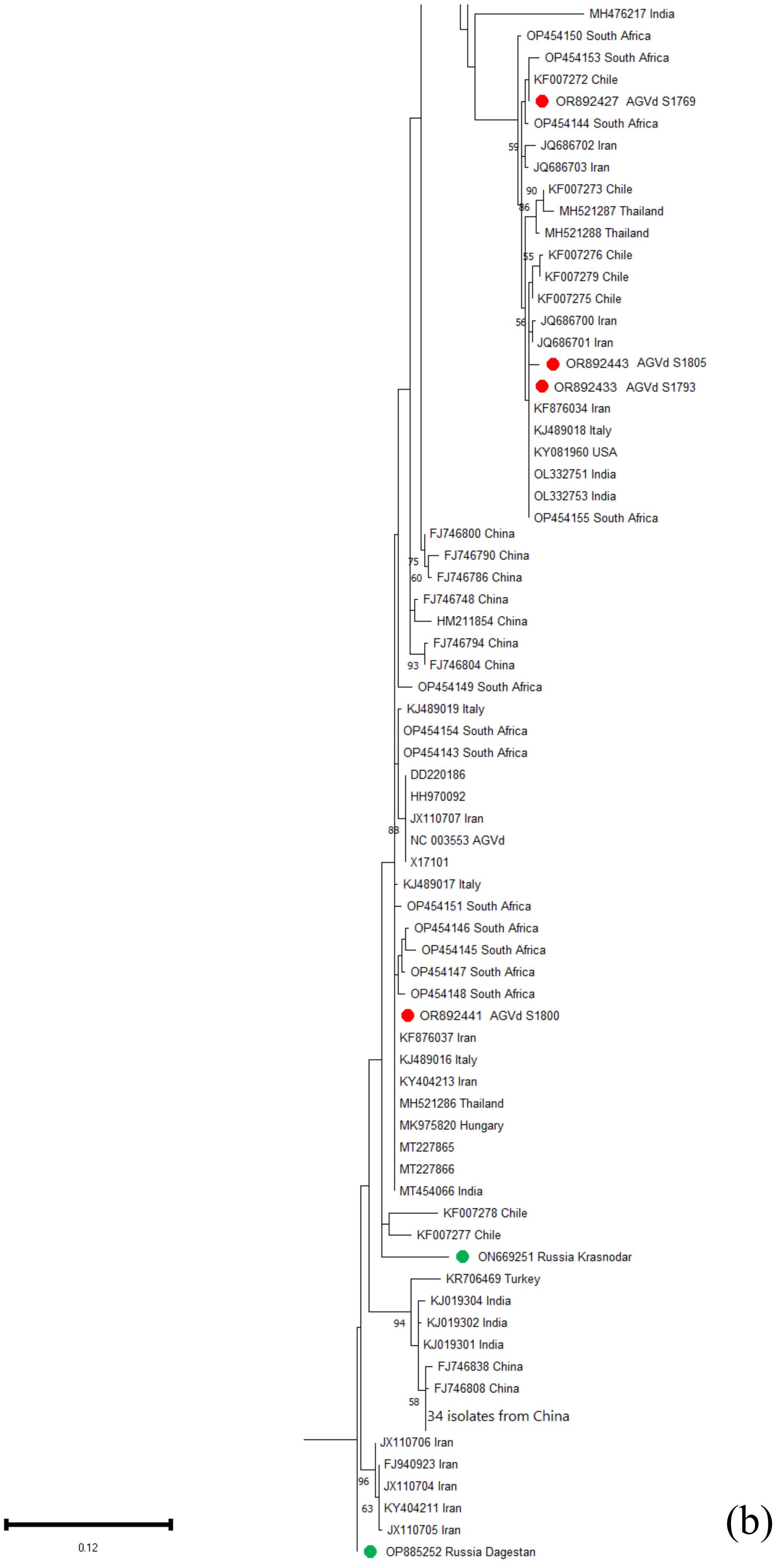


**Supplementary Figure S20 (a-b).** Phylogenetic tree based on complete genome sequences of australian grapevine viroid (AGVd) isolates obtained in this study (red dots), other Russian isolates (green dots) and world isolates. Tree was constructed in MEGA11 using the maximum-likelihood method and Tamura 3-parameter model with 1000 bootstrap replicates. Bootstrap values below 50 are not shown.

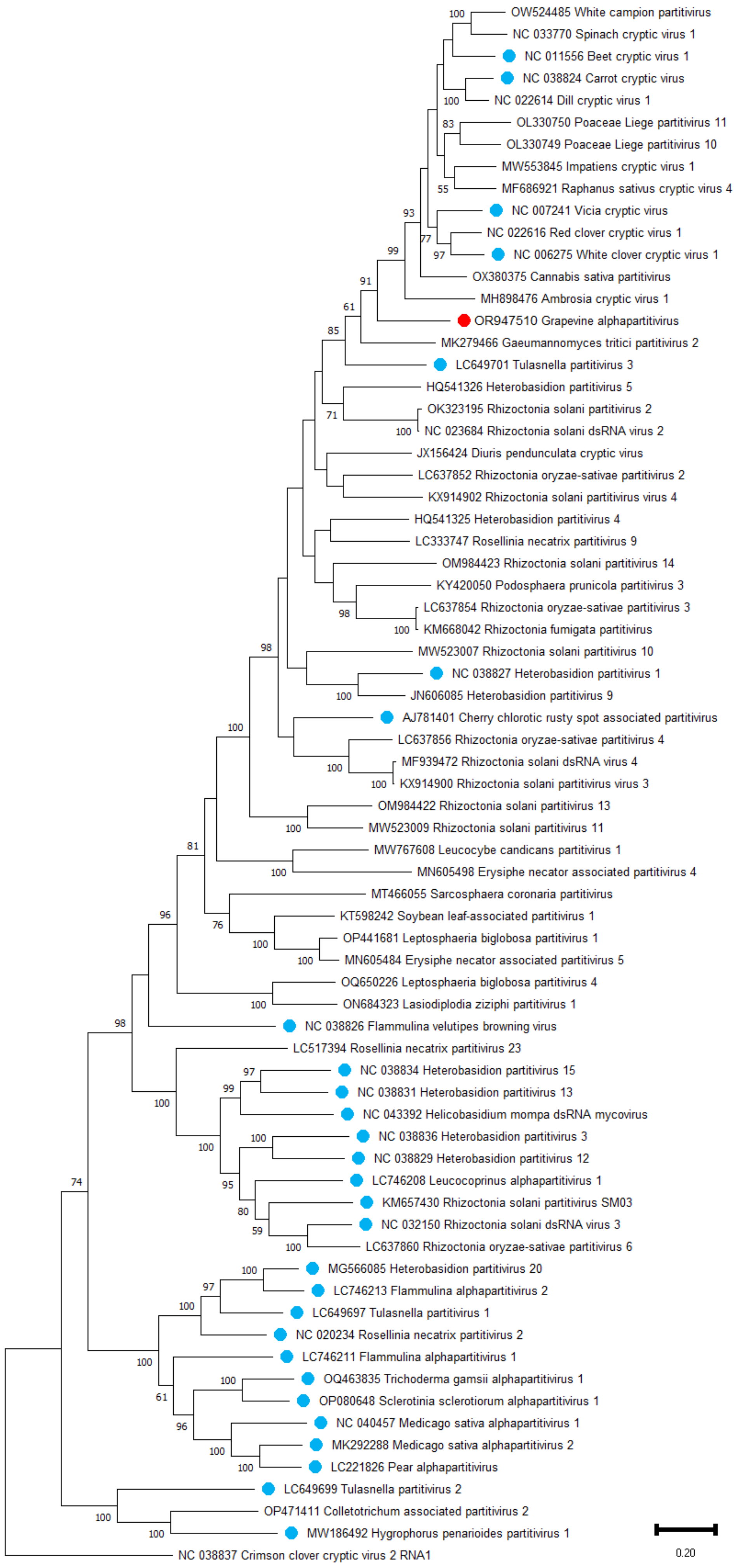


**Supplementary Figure S21.** Phylogenetic tree based on complete nucleotide sequences of RNA1 of GAPV (red dot), alphapartitiviruses (blue dots) and unclassified partitiviruses. Tree was constructed in MEGA11 using the maximum-likelihood method and GTR model with 1000 bootstrap replicates. Boot-strap values below 50 are not shown.

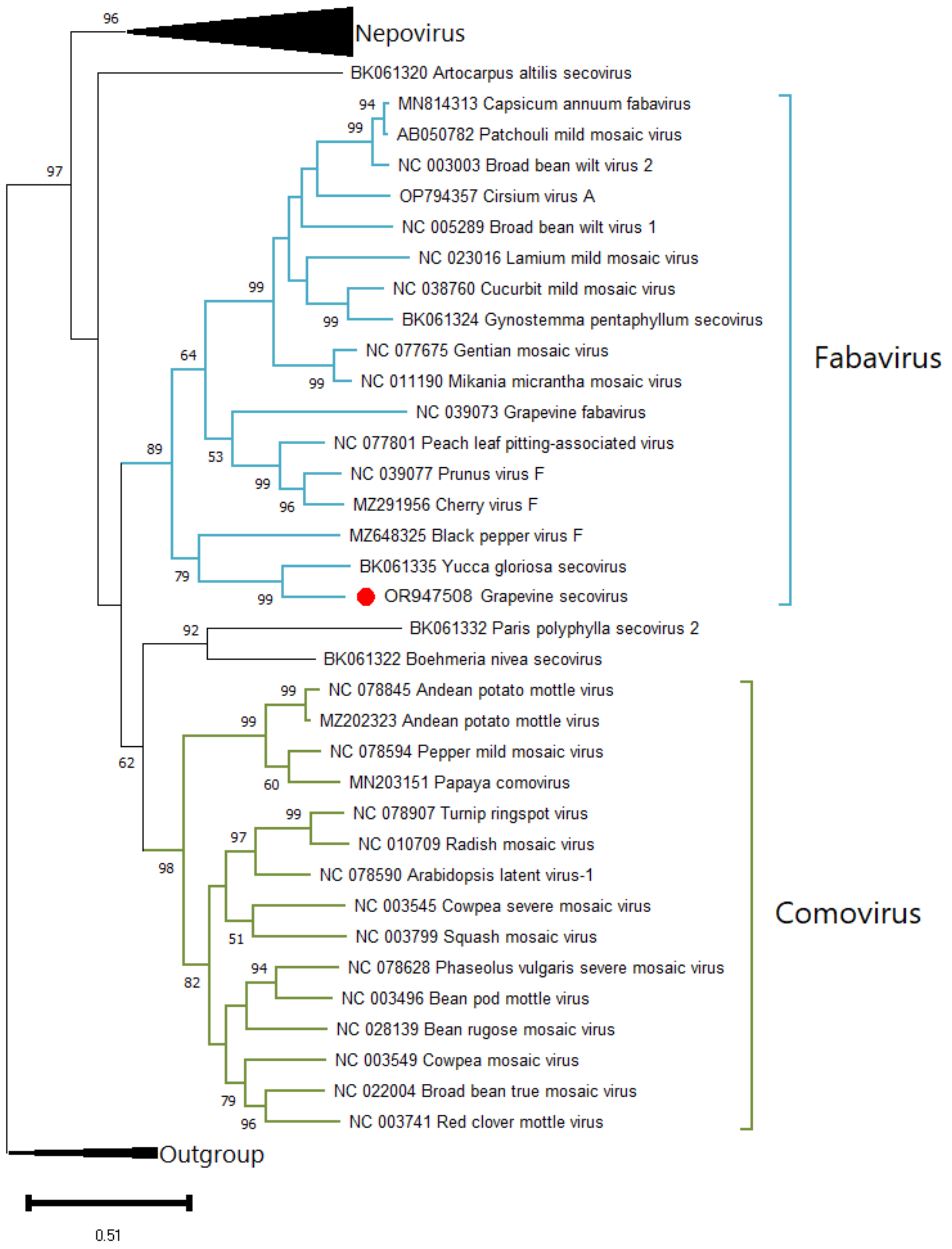


**Supplementary Figure S22.** Phylogenetic tree based on amino acid sequences of the Pro-Pol region of GSV (red dot) and members of the genera *Nepovirus, Fabavirus, Comovirus*. Tree was constructed in MEGA11 using the maximum-likelihood method and GTR model with 1000 bootstrap replicates. Boot-strap values below 50 are not shown.

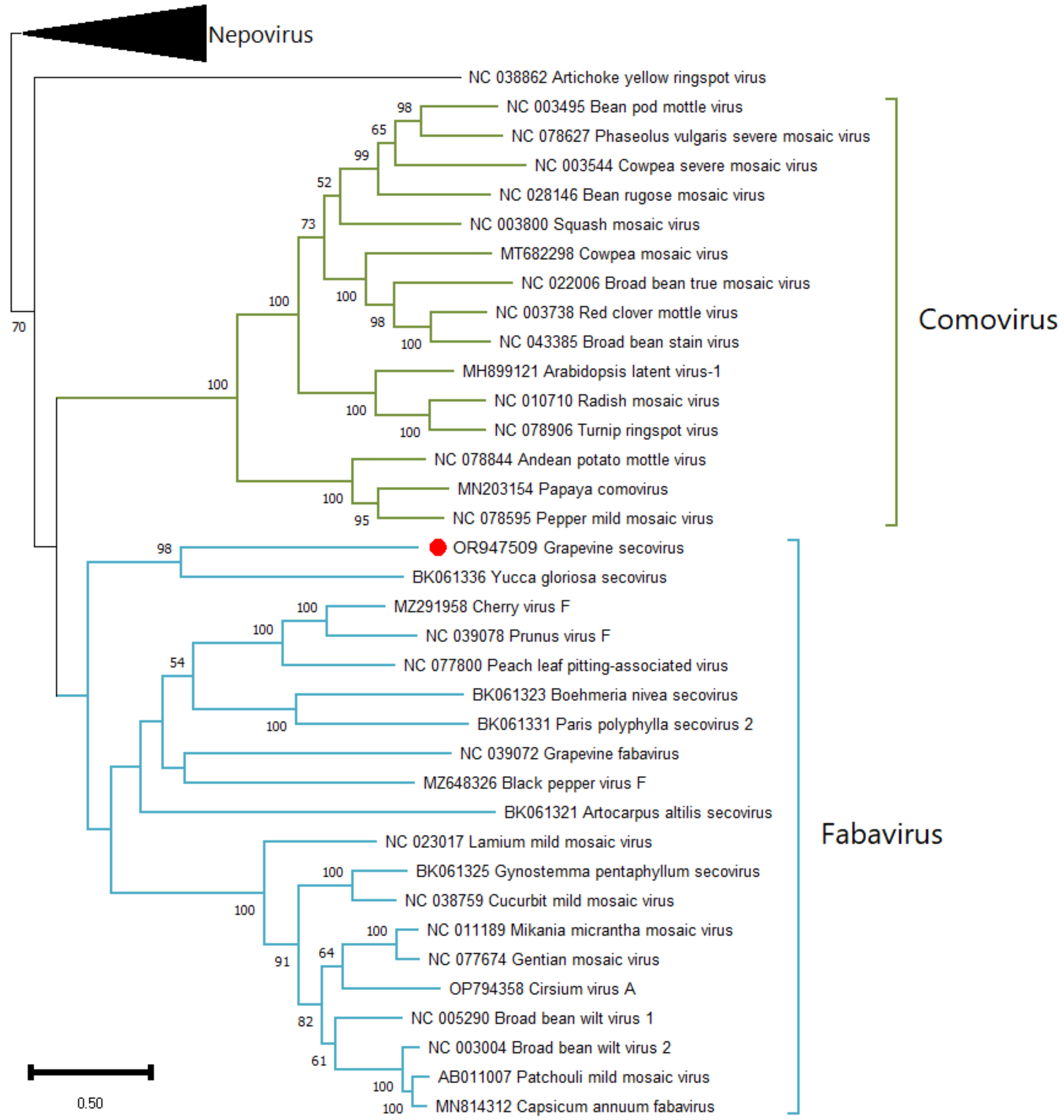


**Supplementary Figure S23.** Phylogenetic tree based on CP amino acid sequences of GSV (red dot) and members of the genera *Nepovirus, Fabavirus, Comovirus*. Tree was constructed in MEGA11 using the maximum-likelihood method and GTR model with 1000 bootstrap replicates. Bootstrap values below 50 are not shown.

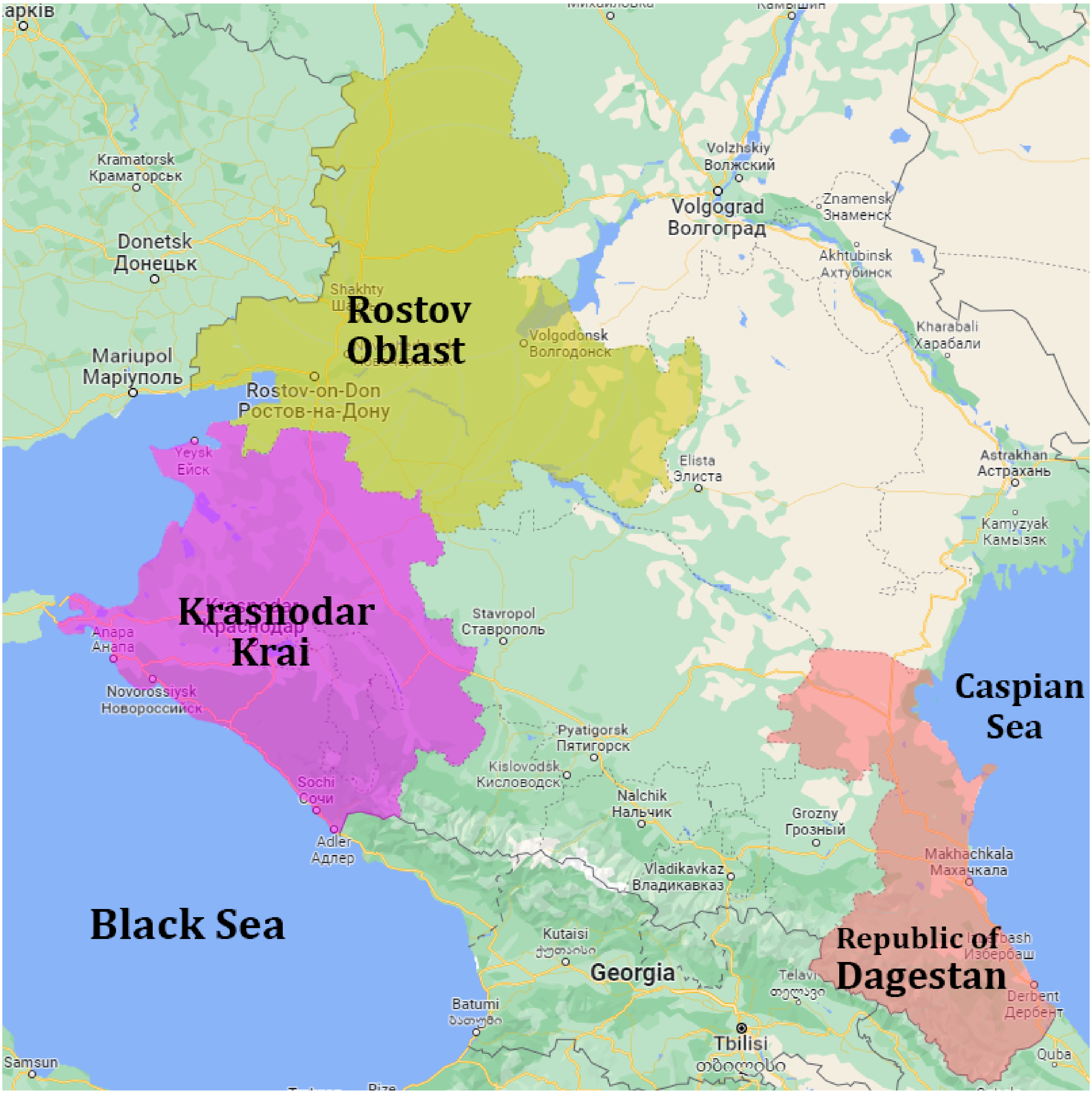


**Supplementary Figure S24.** Map of the southern regions of Russia, where metaviromic studies of ampe-lographic collections were conducted.
